# Functional analysis of LIPID TRANSFER PROTEIN 6 (LTP6) in pennycress and Arabidopsis reveals divergent roles in oil storage and seed coat development

**DOI:** 10.1111/tpj.71038

**Published:** 2026-07-13

**Authors:** Athanas Guzha, Julius Ver Sagun, Christopher Johnston, Payton Whitehead, Ana Paula Alonso, Kent D. Chapman

**Affiliations:** ^1^ BioDiscovery Institute and Department of Biological Sciences University of North Texas Denton Texas USA; ^2^ Present address: Department of Botany and Plant Pathology Purdue University West Lafayette Indiana USA

**Keywords:** embryos, endoplasmic reticulum, lipid droplets, mucilage, pennycress, seed coat, seed germination

## Abstract

In angiosperms, lipid transfer proteins (LTPs) perform multiple roles, including the shuttling of lipids between organelles and to/through the apoplast. In pennycress (*Thlaspi arvense* L.), the LIPID TRANSFER PROTEIN 6 (TaLTP6) was identified as highly expressed in developing embryos, especially in high‐oil accessions. Ectopic expression of pennycress *LTP6* (*TaLTP6*) in *Nicotiana benthamiana* and *Arabidopsis thaliana* leaf mesophyll cells induced the proliferation of cytoplasmic lipid droplets (LDs), suggesting a role in neutral lipid accumulation. GFP‐tagged TaLTP6 localized predominantly to LDs and endoplasmic reticulum (ER)/LD contact sites, while its Arabidopsis homolog, AtLTP6, localized to the ER and apoplast. Domain‐swapping experiments revealed that their N‐terminal regions determined these subcellular localizations. Loss‐of‐function mutants of Arabidopsis *ltp6* exhibited major disruptions in LD organization in mature embryos, characterized by large lipid aggregates, and reduced seed oil content. Proteomics analysis revealed mislocalization of LD‐ and ER‐associated proteins in *ltp6* mutants, suggesting impaired LD biogenesis. Further, Arabidopsis *ltp6* seeds exhibited reduced mucilage extrusion and impaired germination, pointing to a secondary role for AtLTP6 in seed coat function. Complementation of Arabidopsis *ltp6* with *TaLTP6* restored LD morphology and seed oil levels, but did not rescue mucilage and germination defects, indicating functional divergence between the two homologs. We conclude that LTP6 plays a dual role in seeds: (1) participation in embryo lipid storage, and (2) contribution to seed coat integrity and germination. The embryo‐specific expression of *TaLTP6* in pennycress suggests that it retained its evolutionary role in lipid storage, but lost functions related to seed coat development and germination.

## Significance Statement

Many proteins are highly correlated with oil content in mature seeds of pennycress, including a lipid transfer protein (LTP6), and its ectopic production in *Nicotiana benthamiana* increases the abundance of cytosolic lipid droplets. LTP6 loss‐of‐function mutations in Arabidopsis showed disruption in both seed lipid storage and seed coat integrity, and only seed storage was ameliorated by expressing pennycress LTP6, suggesting incomplete conservation of function by LTP6 across these two *Brassica* species.

## INTRODUCTION

Non‐specific lipid transfer proteins (LTPs) are known to shuttle various lipids in a non‐vesicular manner between different membranes (Kader, 1996; Wong et al., 2019). LTPs constitute a large, complex gene family in plants, and they are characterized as small, soluble proteins that are present in all terrestrial plant species, emphasizing their importance for plant function (Boutrot et al., 2008; Edqvist et al., 2018; Salminen et al., 2016; Wei & Zhong, 2014). The recent discovery of LTPs in green algae (Huang et al., 2023) suggests that these proteins evolved prior to terrestrial colonization.

LTP protein structures generally consist of four to five α‐helices formed by a conserved eight‐cysteine motif that forms a hydrophobic cavity for binding to lipid moieties, such as fatty acids (FA), fatty acyl‐CoAs, and phospholipids (Edqvist et al., 2018; Gao et al., 2022). The conserved cysteine residues, C‐Xn‐C‐Xn‐CC‐Xn‐CXC‐Xn‐C‐Xn‐C, form disulfide bridges that stabilize the hydrophobic, lipid‐binding cavity (Melnikova et al., 2018; Missaoui et al., 2022). In addition, most LTPs contain an N‐terminal signal peptide directing the proteins to the secretory pathway (Edstam et al., 2011). While animal LTPs show a more restricted specificity for their substrates (Lev, 2010), plant LTPs often exhibit non‐specific binding activity to different lipids (Kader, 1996). The highly regulated expression, subcellular localization, and evolution of LTPs are all relevant to the widespread function of this protein family in plant development and adaptation to various environmental conditions (Missaoui et al., 2022).

One of the earliest functions characterized for LTPs was a role in plant defense against pathogens, resulting in their inclusion in the pathogenesis‐related protein 14 (PR14) family (Sels et al., 2008). LTPs have been implicated in many other important functions, such as cuticle formation, seed germination, response to abiotic stress, and reproductive development (Debono et al., 2009; Fang et al., 2023; Jacq et al., 2017; Pagnussat et al., 2015; Philippe et al., 2021; Wan et al., 2020; Xu et al., 2018). However, gaps remain in a complete understanding of the varied and complex roles for LTPs in lipid metabolism, trafficking, and homeostasis. Recently, *LTP* transcripts were reported in developing seeds of *Brassica napus*, which suggests the possibility that LTPs participate in the accumulation of storage oil (Chen et al., 2022) and may represent an opportunity to improve oil properties in oilseed crops.

Like other oilseeds, pennycress seeds accumulate storage lipids in the form of triacylglycerols (TAGs) which serve as a primary source of energy for seed germination and early seedling establishment. Seed oil derived from pennycress is dominated by the monounsaturated very long chain fatty acid (VLCFA) erucic acid (C22:1) that constitutes up to 50% of total seed FAs (Altendorf et al., 2019; Arias et al., 2023; Romsdahl et al., 2022), making it an appealing, renewable source of oils for industrial uses (Berman, 2024) and aviation biofuels (Mousavi‐Avval & Shah, 2021; Phippen et al., 2022; Romsdahl et al., 2022).

Lipid droplets (LDs) are the primary neutral lipid storage organelle in seeds and are distributed throughout the embryos of most oilseeds (Guzha et al., 2023; Lujan et al., 2025; Scholz et al., 2022). The LD compartment is unique, consisting of a phospholipid monolayer (instead of a lipid bilayer) derived from the endoplasmic reticulum (ER), which is the principal site of LD biogenesis. The core of LDs consists mainly of TAGs as well as other neutral lipids, such as sterol esters, wax esters, etc. (Guzha et al., 2023; Ischebeck et al., 2020; Scholz et al., 2022). The surface of LDs is embedded with proteins that are important for LD stability, turnover, and other functions. In seeds, oleosins are the most abundant structural LD proteins, and they play a crucial role in LD stability, especially during seed desiccation and rehydration (Huang, 2018; Miquel et al., 2014). While the full catalog of LD‐associated proteins in pennycress seeds has not been fully elucidated, recent work has shown that many LD‐associated proteins identified in Arabidopsis seeds are also present in the pennycress LD proteome. Among the LD proteins identified in pennycress are LIPID DROPLET‐ASSOCIATED PROTEIN 1 (LDAP1), LIPID DROPLET‐ASSOCIATED PROTEIN 2 (LDAP2), LIPID DROPLET‐ASSOCIATED PROTEIN 3 (LDAP3), LIPID DROPLET‐ASSOCIATED PROTEIN [LDAP]‐INTERACTING PROTEIN (LDIP), STEROLEOSIN1, and CALEOSIN1 (Guzha et al., 2024; Lujan et al., 2025). The function of pennycress LD‐associated proteins is likely conserved across the two plant species (Guzha et al., 2024). For example, disruption of pennycress LDIP resulted in the formation of supersized LDs in mutant seeds, which is a phenomenon that was previously identified in Arabidopsis *ldip* mutants (Guzha et al., 2024; Pyc et al., 2021).

To obtain a better molecular understanding of oil yield in pennycress seeds, we recently completed and reported an unbiased transcriptome‐wide study of embryos at four developmental stages derived from 22 pennycress accessions with varying oil content (Arias et al., 2023; Guzha et al., 2024). Numerous LD‐related proteins were identified where transcript abundance was correlated with oil content in mature seeds. Surprisingly, one of the most abundant transcripts in developing embryos was a non‐specific *LIPID TRANSFER PROTEIN 6* (*TaLTP6*). A more detailed expression analysis using two accessions, one with high‐oil yield (TAMN106) and another with low‐oil yield (Ames 32872) confirmed the greater level of expression of *TaLTP6* in the high‐oil accession along with other proteins involved in storage lipid production. Because there was a lack of functional information for LTP6 in developing seeds, we explored in more detail here the role of LTP6 in seed oil accumulation, both in pennycress and in its close relative, *Arabidopsis thaliana*. Our results reveal new perspectives for LTPs in storage lipid accumulation.

## RESULTS

### *TaLTP6* is highly expressed in high‐oil pennycress and shares predicted structural features with Arabidopsis AtLTP6

**Figure 1:**
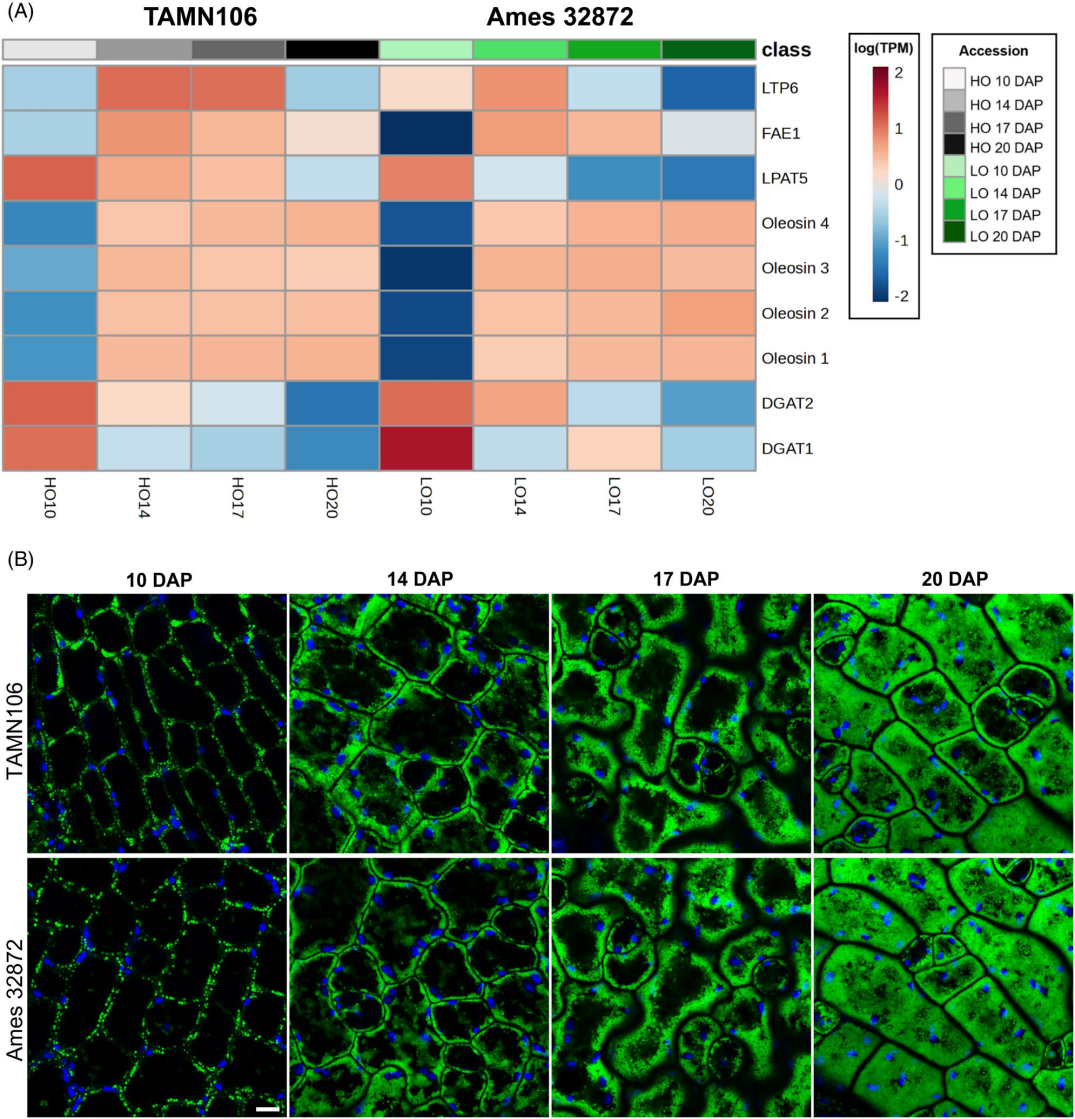
Pennycress LTP6 is highly expressed in developing pennycress embryos. (A) Heat map diagram showing relative transcript abundance of pennycress LTP6 and other lipid associated proteins, FAE1, LPAT5, DGAT1, DGAT2, OLEOSIN1, OLEOSIN2, OLEOSIN3, and OLEOSIN4 in the developing embryos of two pennycress accessions. The expression levels of the proteins were measured using three replicates of developing pennycress embryos at 10, 14, 17, and 20 days after pollination (DAP) using a high‐oil (HO) accession (TAMN106) and a low‐oil (LO) accession (Ames 32872). Red, positive log fold change (log FC) indicates higher expression in the embryo; blue, negative log FC. (B) Representative confocal laser scanning microscopy images of BODIPY‐stained LDs (green) in cotyledon tissues of pennycress embryos at 10, 14, 17, and 20 DAP derived from a high‐oil accession, PC22 (TAMN106), and a low‐oil accession, PC2 (Ames 32872). Blue is autofluorescence from chlorophyll. Bar = 5 μm.

The previous published transcriptome analysis performed by members of our group using various pennycress accessions revealed the expression of numerous genes related to lipid production (Arias et al., 2023; Guzha et al., 2024). Among the genes showing highest expression was *TaLTP6*, and its expression was quantified in the high‐oil pennycress accession (TAMN106) and the low‐oil pennycress accession (Ames 32872), which are accessions with similar plant developmental rates yet significantly different oil yield (oil contents quantified in Figure S1). *TaLTP6* was highly expressed in both lines, but the expression was higher in the high‐oil accession, especially at 14 and 17 days after pollination (DAP; Figure 1A). For comparison, the expression of several other genes involved in storage lipid accumulation in pennycress embryos was analyzed, including *FATTY ACID ELONGATION1* (*FAE1*, AT4G34520), *LYSOPHOSPHATIDIC ACID ACYLTRANSFERASE5* (*LPAT5*, AT3G18850), *DIACYLGLYCEROL ACYLTRANSFERASE1* (*DGAT1*, AT2G19450), *DIACYLGLYCEROL ACYLTRANSFERASE2* (*DGAT2*, AT3G51520), and the oleosins (*OLEOSIN1* AT4G25140, *OLEOSIN2* AT5G40420, *OLEOSIN3* AT5G51210, and *OLEOSIN4* AT3G27660). As expected, these lipid‐related genes were highly expressed during embryo development (Figure 1A). *TaLTP6* expression increased at 14 and 17 DAP along with *FAE1* and *LPAT5*. *DGAT1* and *DGAT2* were highly expressed during early embryo development while the expression of oleosins increased at the late stages of pennycress embryo development (Figure 1A). Pennycress embryos stained for neutral lipids at 10, 14, 17, and 20 DAP provided a visual readout of LD abundance and neutral lipid accumulation during embryo development (BODIPY, green fluorescence; Figure 1B).

**Figure 2:**
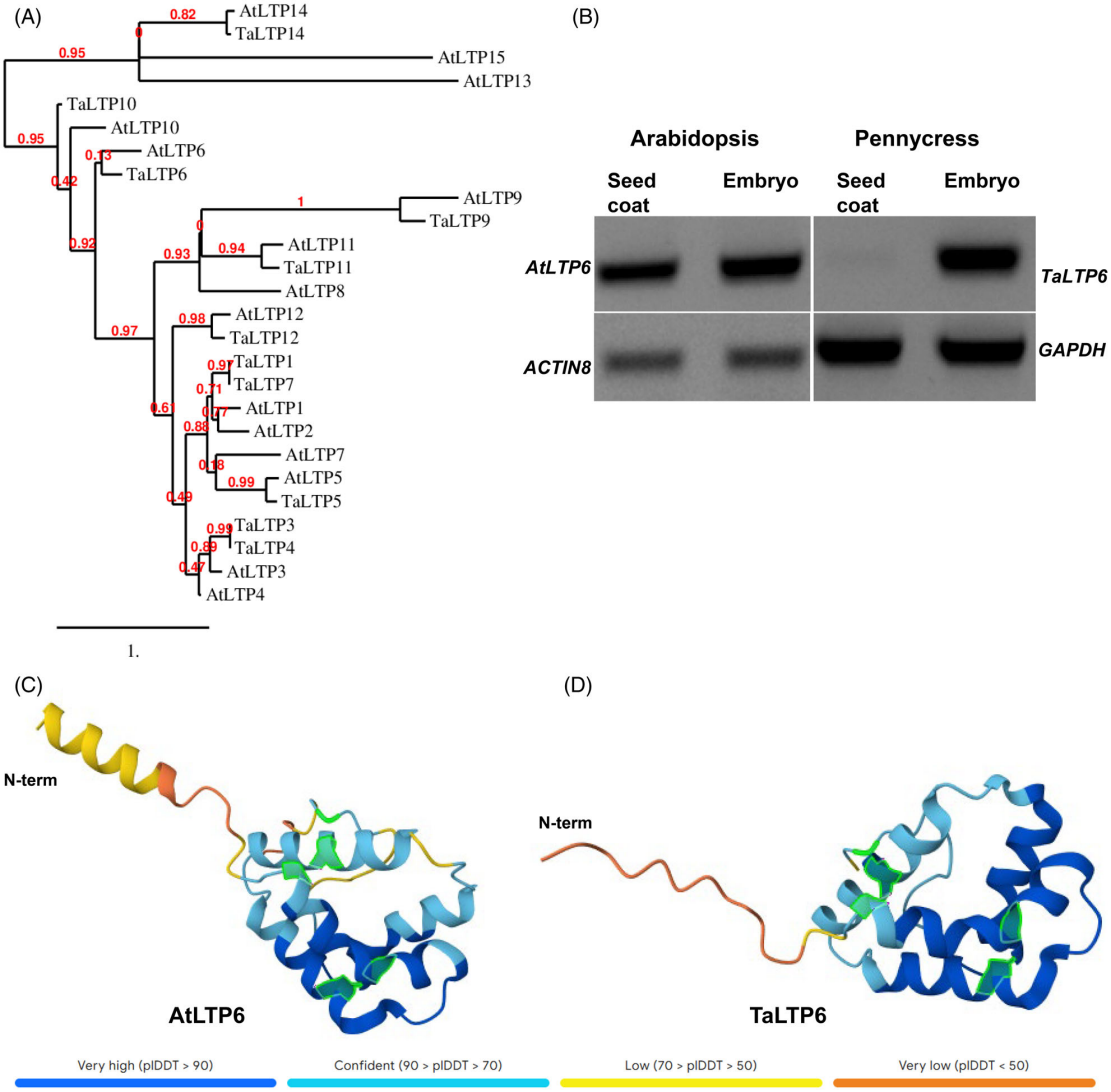
Comparative analysis of LTP6 in pennycress and Arabidopsis. (A) Phylogenetic tree of pennycress lipid transfer proteins (LTPs) identified in embryos with PR14 (pathogenesis‐related) family in Arabidopsis. The evolutionary history was inferred by using the maximum likelihood method based on the JTT matrix‐based model (Jones et al., 1992). Scale bar, 1 number of substitutions per site. (B) Reverse transcription polymerase chain reaction (RT‐PCR) showing the expression of Arabidopsis and pennycress LTP6 in the embryos and seed coat. Arabidopsis LTP6 is expressed at the same levels in the embryos and the seed coat while in pennycress the expression is mostly confined to the embryos. (C, D). Alphafold 3 models of pennycress and Arabidopsis LTP6 showing confidence‐per‐residue coloring (pLDDT) and domain organization. Structures are shown in ribbon‐diagram representation. Residues highlighted in green are the conserved cysteine residues forming disulfide bridges. The predicted general protein structure is mostly conserved across the two plant species.

Because pennycress is a close relative of Arabidopsis (Chopra et al., 2018), we compared the expression of LTP6 in the seeds of the two species. Interestingly, *TaLTP6* was expressed in embryos exclusively, whereas *AtLTP6* was expressed in both embryos and seed coat tissues (Figure 2B). Our reverse transcription polymerase chain reaction (RT‐PCR) results were consistent for *AtLTP6* where publicly available databases (eFP browser, https://bar.utoronto.ca/efp_arabidopsis/cgi‐bin/efpWeb.cgi) indicated that *AtLTP6* was expressed in developing seeds and other plant parts/organs (Figure S3A). Public databases also show that *AtLTP6* is highly expressed in developing embryos as well as the outer integument of the developing seeds (Figure S3B,C). The evolutionary relatedness of TaLTP6 to AtLTP6 was determined by phylogeny analysis. A phylogenetic tree derived from the amino acid sequences of pennycress LTPs detected in the embryo transcriptome data and members of the Arabidopsis Pathogenesis‐Related family 14 (PR‐14; Sels et al., 2008) to which AtLTP6 belongs, revealed that TaLTP6 clustered together with its AtLTP6 ortholog as expected (Figure 2A). *In silico* analysis of TaLTP6 and members of the PR‐14 family indicated that most members were predicted to contain an N‐terminal signal peptide for trafficking to the secretory pathway (SignalP software; Teufel et al., 2022; Figure S2A–C).

To gain further insight into the similarity of TaLTP6 to AtLTP6, we examined their predicted protein structure models generated by the Alphafold 3 software (Abramson et al., 2024). The two orthologs revealed the presence of four conserved α‐helices in the middle of the protein, like other known LTPs (Deeken et al., 2016; Gincel et al., 1994; Safi et al., 2015). The N‐terminus of AtLTP6 was predicted, albeit with less confidence (pLDDT <70), to form an α‐helix while the C‐terminus was less organized (Figure 2C). In pennycress, both the N‐ and C‐termini had no secondary structure predicted, although these protein segments were modeled with lower confidence (pLDDT <70; Figure 2D). Hydropathy plots for both proteins revealed the close similarity, as they both possess a hydrophobic region in the N‐terminus, which was also predicted to be a signal peptide (Figure S2D,E). No putative hydrophobic transmembrane‐spanning sequences were noted (Figure S4A,B). Helical wheel (Gautier et al., 2008) projections generated using the N‐terminus of both proteins revealed no major differences between them (Figure S4C,D). Detailed *in silico* analysis of the four conserved α‐helices in the middle of the LTP6 protein using BioLip2 (Zhang et al., 2024) revealed that they formed a hydrophobic cavity capable of binding to LCFA and potentially VLCFA. Lipid‐binding model predictions show that both LTP6 orthologs can bind stearic acid for instance (C18; Figure S5) and the hydrophobic cavity is flexible enough to bind other lipid substrates, including phospholipids.

### TaLTP6 promotes LD proliferation and storage lipid accumulation in leaves

**Figure 3:**
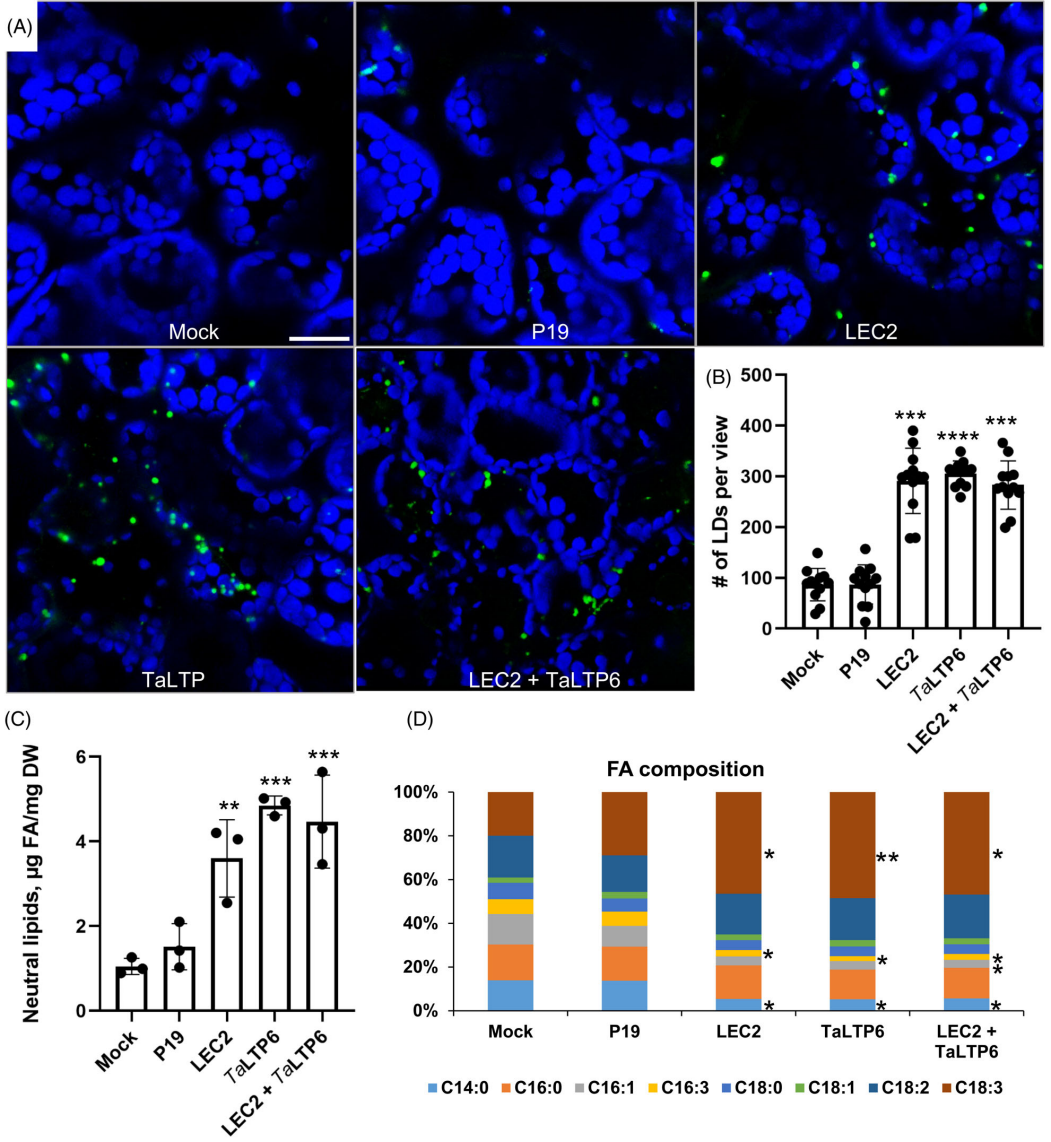
Overexpression of pennycress LTP6 induced a proliferation of LDs in leaves of *Nicotiana benthamiana* and alters the fatty acid (FA) composition. (A) Representative confocal laser scanning microscopy images (*z*‐stacks) of LD abundance in *N. benthamiana* leaves showing that the ectopic overexpression of pennycress LTP6 (*Ta*LTP6) coding sequence resulted in the increased abundance of LDs. LDs are stained (green) with BODIPY while blue is autofluorescence from the chloroplasts. Bars = 20 μm. (B) The LD abundance in infiltrated *N. benthamiana* leaves was quantified. Values are averages of three individual experiments with at least three images for each replicate. Error bars show SD. Statistical differences to the mock were determined for each construct (***P < 0.001, ****P < 0.0001, Kruskal–Wallis test followed by Dunn's test). (C) Neutral lipid content in the *N. benthamiana* leaves transformed with TaLTP6 is also shown. Values are averages from three leaves per line. Error bars show SD. Statistical differences to the mock were determined for each construct (**P < 0.01, ***P < 0.001, Kruskal–Wallis test followed by Dunn's test). (D) Variation in the FA profiles for the infiltrated *N. benthamiana* leaves. Statistical differences to the mock were determined for each FA species (*P < 0.05, **P < 0.01, Kruskal–Wallis test followed by Dunn's test).

The high abundance of *TaLTP6* transcripts in developing embryos compelled us to test whether the protein could support biosynthesis and/or compartmentation of storage lipids in plant cells independently. Indeed, transient expression of *TaLTP6* induced LD and storage lipid accumulation in *Nicotiana benthamiana* leaves (Figure 3). For comparison, expression of the transcription factor, *LEAFY COTYLEDON2* (*AtLEC2*) which regulates many lipid accumulation factors in embryos (Stone et al., 2001), increased numbers of LDs and neutral lipid content to a similar extent as TaLTP6 (Figure 3A–C). The ectopic expression of *TaLTP6* under the control of *CaMV35S* promoter induced visual increases in the abundance of cytoplasmic LDs in the mesophyll cells of *N. benthamiana* (Figure 3A). Quantification of the LDs and neutral lipids in infiltrated *N. benthamiana* leaves revealed a similar pattern, with a threefold and sixfold increase in LD numbers and neutral lipid content, respectively, upon *TaLTP6* expression (Figure 3B,C). The expression of *AtLEC2* alone and the co‐expression of *TaLTP6* with *AtLEC2* each increased LD abundance and neutral lipid content to a similar extent as the expression of *TaLTP6* alone and were significantly higher than mock‐infected and P19‐infiltrated (suppressor of transgene silencing) controls. The FA composition of the neutral lipids extracted from infiltrated *N. benthamiana* was altered by these lipogenic proteins (Figure 3D). There was a general, significant increase in the relative abundances of C18:3 FA and a decrease in C14:0 and C16:3 FAs, upon expression of *TaLTP6* and/or *AtLEC2*. Overall, these results demonstrated that *TaLTP6* effectively promoted LD formation, storage lipid increases, and lipid composition changes in *N. benthamiana* leaves in a manner similar to the known seed‐specific, lipogenic transcription factor *AtLEC2*. However, these effects, while similar between the two proteins, were not additive when both proteins were combined. Separately, experiments testing the stable overexpression of *TaLTP6* in Arabidopsis plants showed a consistent and similar elevation of LD numbers in leaf mesophyll cells (compared with wild‐type, non‐transformed leaves; Figure S6) to that observed in transient expression assays (Figure 3A), which supports a general role for TaLTP6 in the promotion of LD accumulation in plant cells.

### Disruption of AtLTP6 alters LD morphology and the spatial distribution of LD‐ and ER‐associated proteins

**Figure 4:**
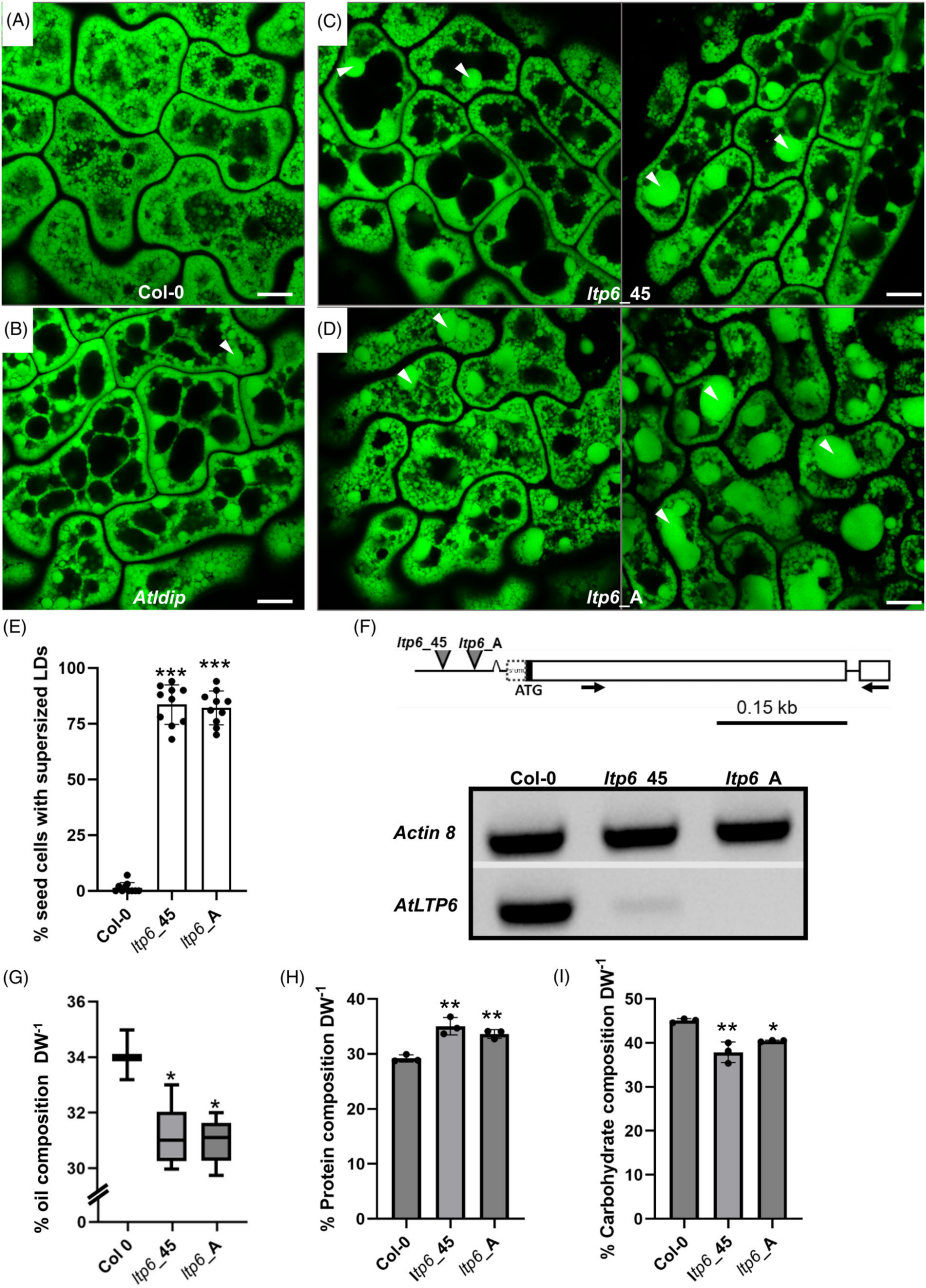
Disruption of LTP6 in Arabidopsis results in the formation of aberrant LDs in mature seeds. Representative confocal laser scanning microscopy images of BODIPY‐stained LDs (in green) in the cotyledons of Arabidopsis dry mature seeds from wild‐type (Col‐0, A), *ldip* mutant (B already shown to produce supersized LDs, white arrows) and two *ltp6* mutant lines (*ltp6*_45 and *ltp6*_A) (C) and (D), respectively. Note the presence of larger sized LDs in the cotyledons of dry mature seeds of *ltp6* mutant lines (C and D, white arrows) after sectioning. (E) Quantification of mature Arabidopsis seed cells containing supersized LDs in cotyledons. Significant differences to the wild‐type (Col‐0) were calculated for each genotype using at least 10 images of seeds derived from 3 plants per genotype (***P < 0.001, Kruskal–Wallis test followed by Dunn's test). (F) Organization of the AtLTP6 (AT3G08770) genomic sequence showing the T‐DNA insertion in the two mutant lines. The arrows show the position of primers used for reverse transcription quantitative polymerase chain reaction (RT‐qPCR). RT‐qPCR analysis indicates that *ltp6*_45 is a strong knockdown while *ltp6*_A is a knockout. (G–I) Oil, protein, and carbohydrate content, respectively, in Arabidopsis seeds derived from Col‐0 and *ltp6* mutants. Error bars show SD. Statistical differences to the wild‐type (Col‐0) were determined for each construct (*P < 0.05, **P < 0.01, Kruskal–Wallis test followed by Dunn's test).

The observed elevation of LDs upon *TaLTP6* ectopic expression posits that the protein impacts storage lipid synthesis, packaging and/or turnover. We, therefore, tested whether loss‐of‐function would influence the production of LDs in seeds. Because of the close sequence similarity between TaLTP6 and AtLTP6, and the lack of mutant resources in pennycress, we obtained two independent Arabidopsis T‐DNA insertion lines for *AtLTP6*. Immediately notable in BODIPY‐stained, mature Arabidopsis seeds were an abundance of very large, malformed LDs in cotyledons of both Arabidopsis mutant lines (*ltp6*_45, SALK_120555 and *ltp6*_A, SALK_04323) compared with the wild‐type background, Col‐0 (Figure 4A,C,D). The large, irregular shapes of the supersized LDs in the Arabidopsis *ltp6* mutants suggested that the LDs were either fusing or that their normal biogenesis process was impaired. As a reference, an Arabidopsis *ldip* mutant (Figure 4B), which also produces supersized LDs (Pyc et al., 2021), was imaged for comparison and showed less severe LD phenotypes than either *ltp6* mutant. Quantification of the presence of aberrant LDs in the cotyledons of mature *ltp6* mutant seeds revealed the dramatic prevalence of these cellular storage lipid defects compared with that in wild‐type (Col‐0; Figure 4E). Both T‐DNA insertions were upstream of the coding DNA sequence of the *LTP6* gene, with one line exhibiting a strong reduction in relative transcript abundance (*ltp6*_45, knockdown), and the other showed essentially no detectable transcript in seed tissues (*ltp6_*A, knockout; Figure 4F). The disrupted LD morphology contributed to a significant reduction in total seed oil content (on a dry weight basis) in both mutants when compared with the wild‐type (Col‐0; Figure 4G). The reduced storage lipid content in the *ltp6* mutants further compelled us to analyze other seed biomass components; both *ltp6* mutants had a significantly higher seed protein content (Figure 4H), while the total seed carbohydrate content was significantly reduced (Figure 4I).

**Figure 5:**
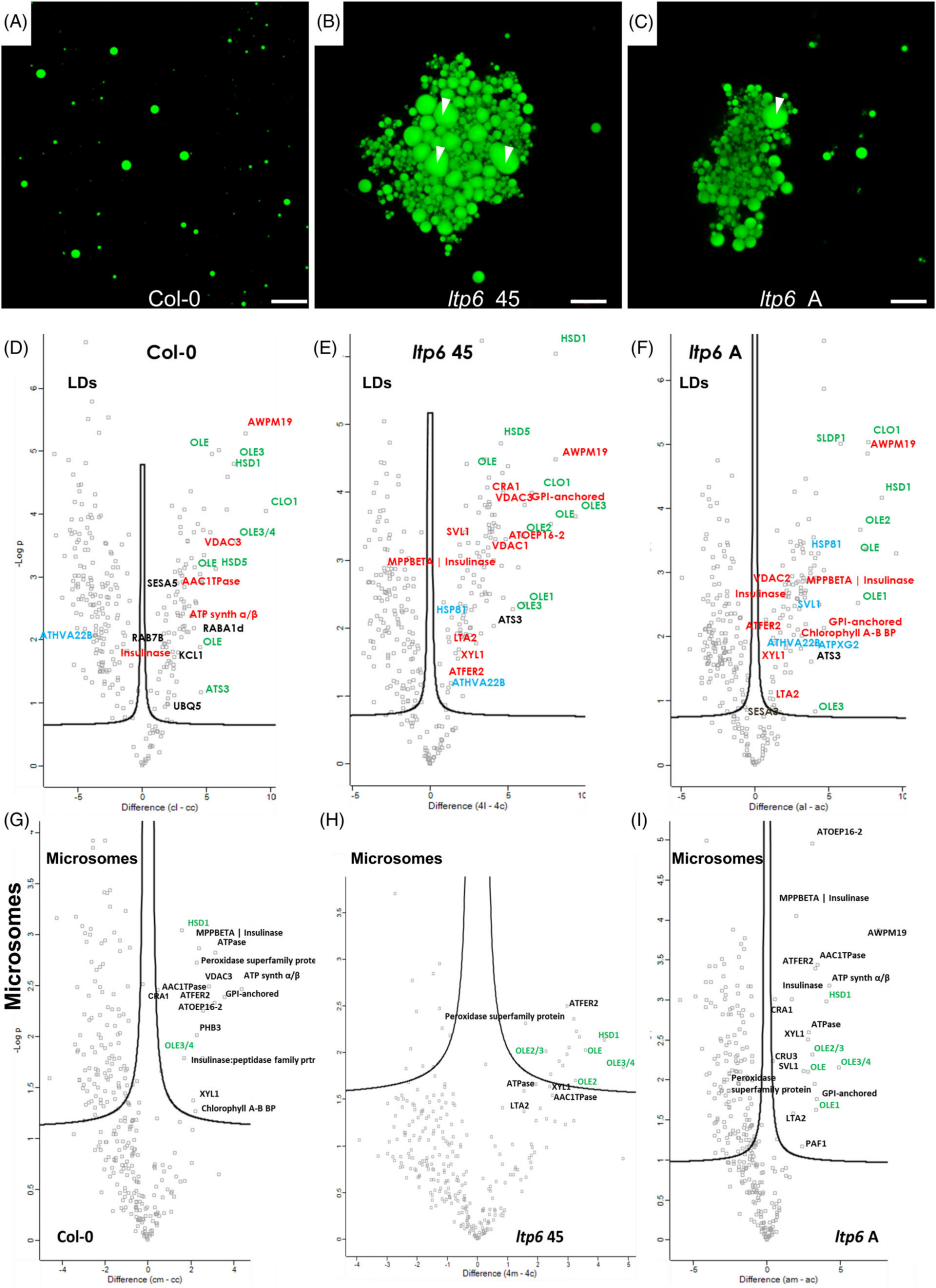
Isolated LDs from seeds of Arabidopsis *ltp6* mutants reveals the presence of large‐sized LDs and increased LD aggregation compared with wild‐type Col‐0. The morphology of the LDs was assessed in isolated LDs from whole dry mature Arabidopsis seeds. The wild‐type, Col‐0 isolated LDs are of uniform size (A), while in the two mutants, large‐sized LDs (white arrows) are observed and there is also a significant increase in the presence of aggregated LDs (B, C). Bars = 10 μm; 20 μm. (D–F) Volcano plots constructed to visualize proteins, which are significantly enriched in the LD fraction of seeds derived from wild‐type (Col‐0), *ltp6*_45 and *ltp6*_A, respectively. The LD‐enriched samples were compared with all total cellular fractions of dry seeds. The log2‐transformed values and *P*‐values were calculated. Known LD proteins are indicated in green, while red represents candidates enriched in the microsomal fractions as shown in (G), (H), and (I). (G–I) Volcano plots constructed to visualize proteins, which are significantly enriched in the microsomal fraction of seeds derived from wild‐type (Col‐0), *ltp6*_45 and *ltp6*_A, respectively. The microsomal‐enriched samples were compared with all total cellular fractions of dry seeds. The log2‐transformed values and *P*‐values were calculated. Known LD proteins are indicated in green. Black lines indicate a false discovery rate of 0.01.

For a more detailed examination of the cellular defects in lipid storage, the LDs of both mutants were isolated from mature Arabidopsis seeds by flotation centrifugation (Horn et al., 2021). The LD morphologies and proteomes were compared, along with the proteomes of microsomes from these same seed samples (Figure 5). Visualization of the isolated, BODIPY‐stained LDs revealed marked aggregates of large‐ and variable‐sized LDs in the mutants, compared with the well‐disbursed uniform sized LDs isolated from wild‐type (Col‐0) seeds (Figure 5A–C). The clustering of these varied‐size LDs observed in the *ltp6* mutants might suggest that the aberrant LDs were tethered to remnants of ER membrane holding them together. This suggests that the normal course of storage lipid ‘filling’ and LD biogenesis in these mutant cells had been disrupted in the absence of LTP6. Isolated LDs always contain some level of contamination from ER‐derived membranes and other hydrophobic proteins, but this can mostly be accounted for by comparing enrichment of LD proteins relative to total cellular proteins (Horn et al., 2021; Kretzschmar et al., 2020; Niemeyer et al., 2022). Here, the proteomes for the LD fractions revealed the enrichment of many structural LD proteins in wild‐type, Col‐0 and both mutants as expected, such as caleosin (CLO1), steroleosins (HSDs), and multiple oleosins (OLEs) (Figure 5D–F, green text; Dataset S1). Interestingly, however, the two mutant lines had considerably more numbers of proteins that were more commonly associated with microsomal fractions that were enriched in these LD fractions (Figure 5E,F, annotated red text; Figure S6C; Dataset S1; see Figure 5G and Table S2 for Col‐0 microsomes, annotated black text), suggesting relatively more membrane contamination of the LD fractions for the *ltp6* mutants. By contrast, microsomal membrane fractions of the *ltp6* mutants revealed an increased abundance of some proteins normally associated with the LDs, including steroleosin (HSD1) and several oleosins (OLEs, annotated in green text) more numerous than the LD proteins found in the microsome fractions of wild‐type Col‐0 (Figure 5G–I). These results could suggest that there is an incomplete scission of LDs from the ER in the absence of LTP6, resulting in more microsomal membrane proteins trapped with isolated LDs, and more LD proteins trapped in microsomes, of the mutants. Although other explanations are possible including possible cross‐contaminations of subcellular fractions, the incomplete subcellular partitioning of LDs from ER in *ltp6* mutants may partially explain the mutant LD phenotypes observed in seeds.

### Aberrant LD phenotypes and germination defects in *ltp6* mutants

**Figure 6:**
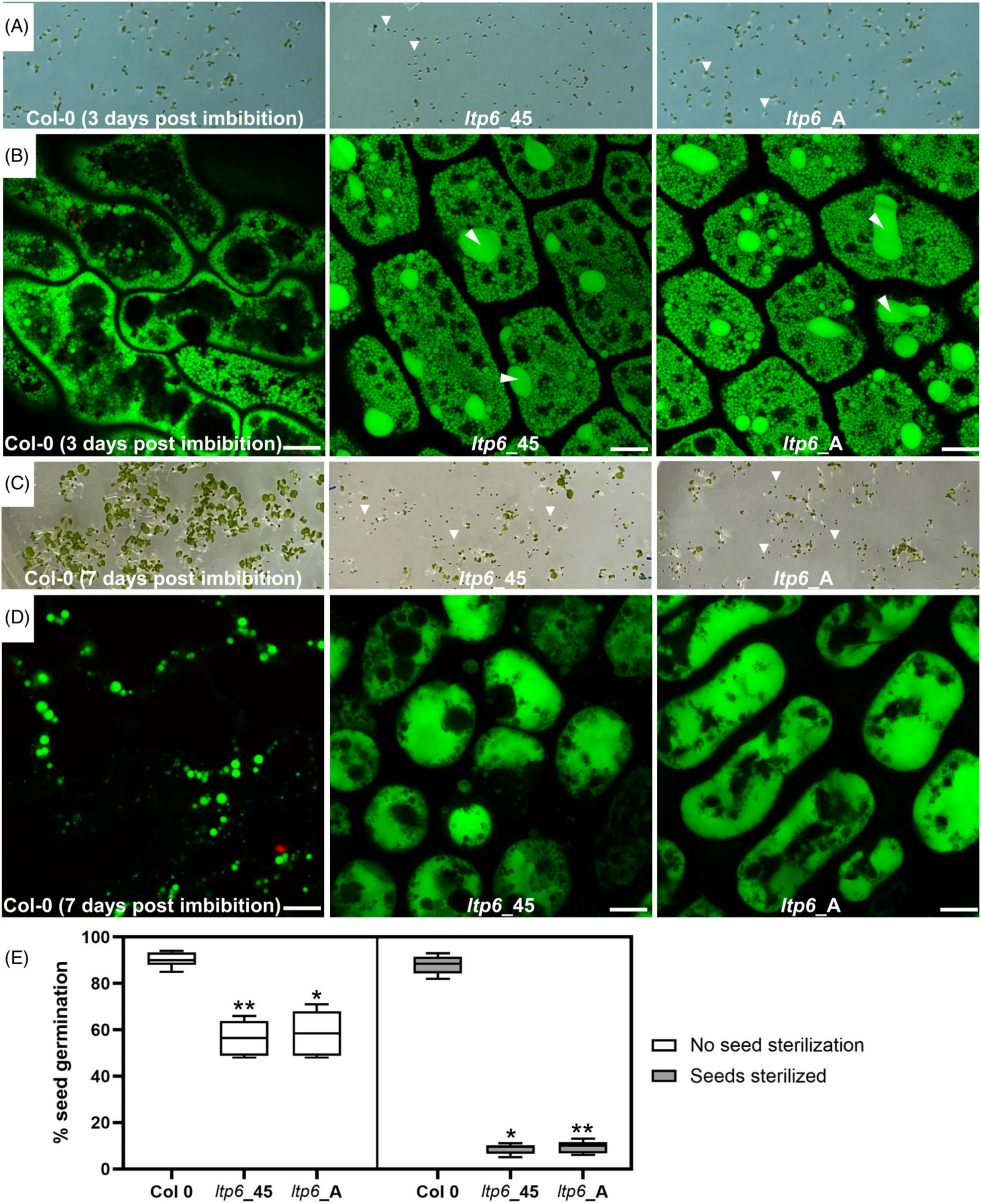
Disruption of LTP6 in Arabidopsis seeds results in reduced germination rates. (A) Germination rates are reduced in the *ltp6* mutants compared with wild‐type Col‐0 at 3 days post‐imbibition. (B) Representative confocal laser scanning microscopy (CLSM) images showing that larger sized LDs (white arrows) persist in the cotyledons of the *ltp6* mutant seeds at 3 days post‐imbibition. (C) At 7 days post‐imbibition most wild‐type Col‐0 seeds have germinated while many mutant seeds fail to germinate. (D) Representative CLSM images showing the loss of compartmentation of lipids in cotyledons of mutant seeds that fail to germinate at 7 days post‐imbibition. Bars = 10 μm. (E) Germination assay for wild‐type and mutant Arabidopsis seeds with and without seed sterilization performed using bleach before plating on growth media. Values are averages derived from 1000 seeds per line split into three replicates. Error bars show SD. Statistical differences to the wild‐type were determined for each genotype (*P < 0.05; **P < 0.01; Kruskal–Wallis test, followed by Dunn's test).

A cellular disruption in storage lipid accumulation in mature embryos might be expected to manifest in reduced germination and seedling growth rate. Indeed, most seeds from the Arabidopsis *ltp6* mutant lines failed to germinate, and those that did had less vigor and grew at slower rates (Figure 6A,C,E; Figure S7). The large BODIPY‐stained LDs were observed in cotyledon cells of both *ltp6* mutants at 3 days post‐imbibition (dpi; Figure 6B) and were especially exacerbated in ungerminated seeds imaged at 7 dpi, where most of the lipids in the seeds lost all form of compartmentation when compared to the wild‐type (Col‐0) that had germinated (Figure 6D).

Sowing seeds directly on soil without any form of seed sterilization showed less impact on seed viability of the *ltp6* mutants, although germination and seedling development were slower in the mutant lines (Figure S7C,D). Therefore, we tested whether the reduced viability was a result of the sterilization process that included treatment with sodium hypochlorite (bleach). Quantification of germination using bleach‐sterilized versus unsterilized seeds revealed that germination was improved considerably in unsterilized seeds for both mutant lines (Figure 6E). While the germination rate and plant growth rates were affected by LTP6 loss‐of‐function, the overall plant morphology at maturity, including plant height, weight, and seed yield, was otherwise unaffected (Figure S8).

To examine whether the susceptibility to seed sterilization with bleach might be due to differences in seed coat surface properties, the amount of extruded mucilage in imbibed seeds was estimated with ruthenium red staining (Figure S9). It was evident that the two Arabidopsis mutant lines shed a lesser amount of mucilage in comparison with the Col‐0 wild‐type seeds, resulting in a reduced amount of adherent mucilage (Figure S9A–C). A comparison of the calculated volumes of adherent mucilage revealed that the wild‐type seeds had a significantly larger volume of adherent mucilage compared with both *ltp6* mutants, which both showed only approximately 50% of the volume of adherent mucilage as that of Col‐0 seeds (Figure S9D).

### GFP‐tagged TaLTP6 and AtLTP6 showed distinctive subcellular localizations in *N. benthamiana* leaves

**Figure 7:**
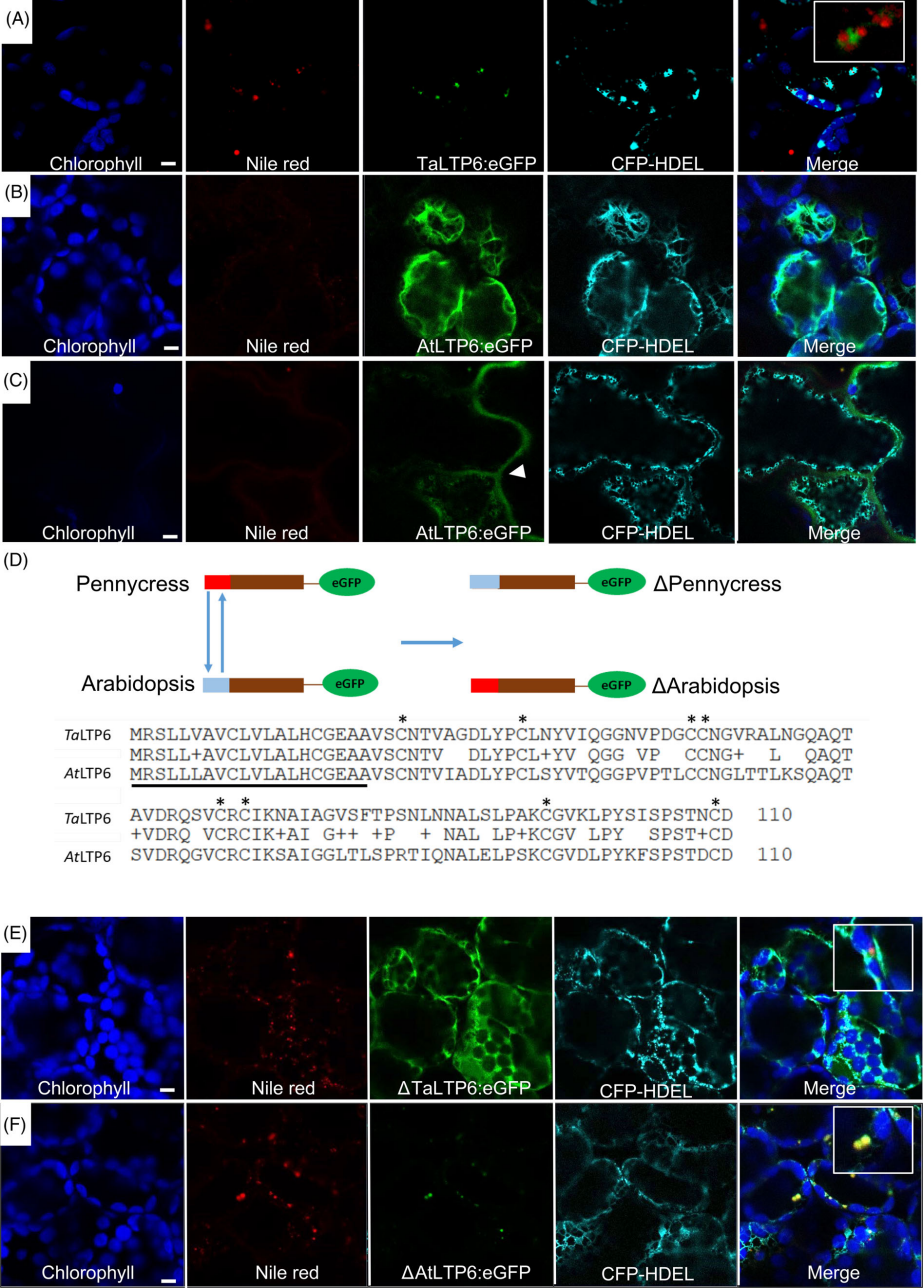
Subcellular localization of pennycress and Arabidopsis LTP6. Representative confocal laser scanning microscopy (CLSM) images (*z*‐sections) of *Nicotiana benthamiana* leaf epidermal cells co‐transformed with TaLTP6:eGFP (A), AtLTP6‐eGFP (B, C) with CFP‐HDEL serving as the endoplasmic reticulum (ER) marker protein and LDs are stained red with Nile red while blue is autofluorescence from the chloroplasts. TaLTP6:eGFP localizes to LDs and LD/ER junctions while AtLTP6:eGFP localizes to ER and apoplast. The arrow (C) shows the presence of GFP fluorescence in the regions where two or more cells meet, suggesting apoplastic localization. (D) The predicted signal peptides of pennycress and Arabidopsis LTP6 were swapped to generate chimeric Arabidopsis and pennycress LTP6 proteins with GFP appended to the C‐terminus. The underlined region in the polypeptide sequence alignment represents the predicted N‐terminus signal peptide while asterisks show conserved cysteine residues. (E) The chimeric TaLTP6 with an Arabidopsis signal peptide localizes mostly to the ER. (F) The chimeric AtLTP6 with a pennycress signal peptide localizes to the LDs. CFP‐HDEL was used as the ER marker protein and LDs are stained red with Nile red while blue is autofluorescence from the chloroplasts. The inserted boxes in some merged images represent a portion of the cells shown at a higher magnification. Bars = 10 μm.

TaLTP6 and AtLTP6 exhibited high sequence identity at the amino acid level, and both were predicted to possess nearly identical putative N‐terminal signal sequences (Figures S2 and S4), although the protein environments around the N‐terminus were predicted to be somewhat different between the alphafold models of the two proteins (Figure 2C,D). Consequently, the subcellular location of these two homologs was tested in transient expression assays in *N. benthamiana* (Figure 7), where TaLTP6 had been shown to promote LD formation (Figure 3). Here, the coding sequences of both TaLTP6 and AtLTP6 with eGFP appended to the C‐terminus of the proteins were expressed as GFP fusions in the leaves of *N. benthamiana*. The TaLTP6‐GFP protein localized mostly to LDs (marked by Nile red) and LD/ER junctions (ER marked by CFP‐HDEL which consists of the KAR2 signal sequence and C‐terminal HDEL ER retrieval signal) (Figure 7A; Figure S10A). It was also observed that the ectopic expression of *TaLTP6* also resulted in a reorganization of the ER, resulting in ER fragments that colocalized with or next to Nile red stained LDs (Figure 7A; Figure S10A). Furthermore, *TaLTP6*, when expressed in stable Arabidopsis transgenic lines, also localized predominantly to LDs (Nile red staining) or adjacent to LDs (Figure S10B). There was no CFP‐marked ER in the stable Arabidopsis lines. By contrast, the AtLTP6‐GFP protein produced transiently in mesophyll cells of *N. benthamiana* leaves localized throughout the ER, coincident with the ER luminal marker, CFP‐HDEL (Figure 7B), whereas the AtLTP6‐GFP produced in epidermal cells of *N. benthamiana* leaves was also detected in regions between cells suggesting apoplastic localization as well as ER (Arrow; Figure 7C). The observed ER and apoplastic localization of AtLTP6 is consistent with the predicted localization available in public databases (Figure S10C). Interestingly, the ectopic expression AtLTP6 hardly induced any formation of cytosolic LDs upon expression in *N. benthamiana* mesophyll cells (Figure 7B,C). Swapping the N‐terminus between these two proteins to generate chimeric LTP6 proteins where the AtLTP6 possessed a pennycress N‐terminus and the TaLTP6 possessed the AtLTP6 N‐terminus (Figure 7D; Figure S10D), switched the subcellular localization of these chimeric proteins; that is, the chimeric TaLTP6 localized to the ER while the chimeric AtLTP6 localized to LDs (Figure 7E,F). These results taken together suggest that these two related proteins have somewhat different subcellular locations and that the N‐terminus influences the differential organelle targeting, either directly or indirectly. Interestingly, the N‐terminus swap experiments also resulted in the proliferation of cytosolic LDs upon expression of both chimeric proteins (Figure 7E,F) in the mesophyll cells of *N. benthamiana*.

### Expression of *TaLTP6* mostly restored the normal LD phenotypes in seeds of both *ltp6* mutants but did not reverse mucilage and germination defects

**Figure 8:**
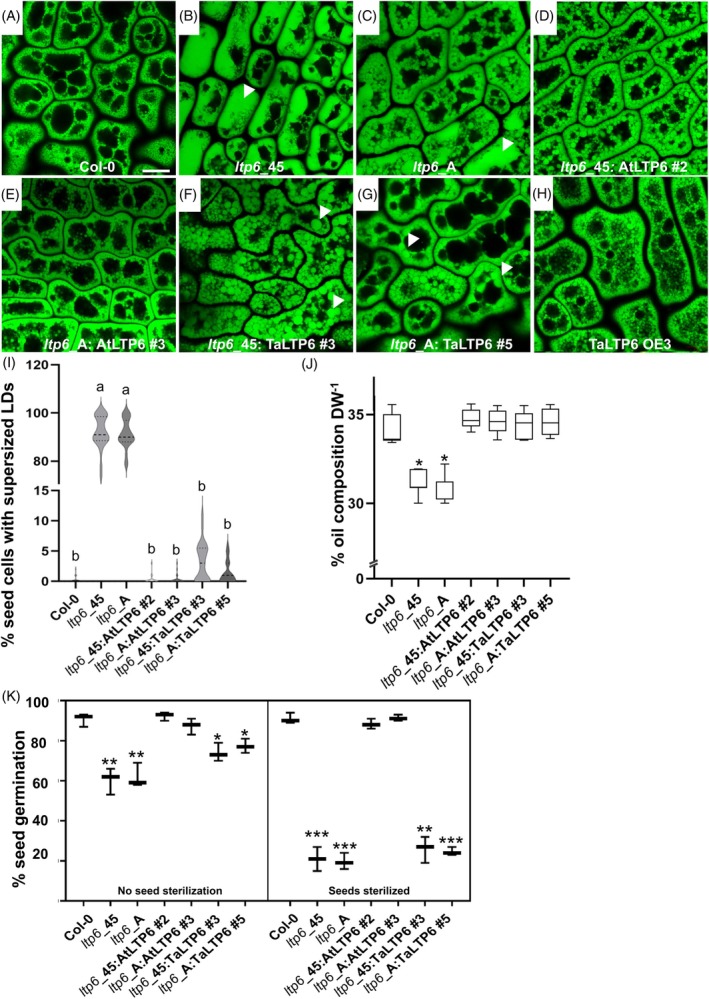
Complementation of Arabidopsis *ltp6* mutants with TaLTP6 partially restores the LDs phenotype. (A) Representative confocal laser scanning microscopy images showing BODIPY‐stained normal LDs (green) in cotyledons of mature seeds from wild‐type Col‐0 (A), and the supersized LDs (white arrows) present in the Arabidopsis *ltp6* mutants (B, C). Representative Arabidopsis *ltp6* lines complemented with AtLTP6 (*ltp6*_45: AtLTP6 #2, *ltp6*_A: AtLTP6 #3) reveal a reversion of phenotype to normal sized LDs similar to Col‐0 (D, E). Transformation of Arabidopsis *ltp6* mutants with TaLTP6 for example *ltp6*_45: TaLTP6 #3, *ltp6*_A: TaLTP6 #5, still results in the presence of large sized LDs (F, G, arrows) although the size of the aberrant LDs is reduced. (H) Representative image showing the overexpression of TaLTP6 (TaLTP6 OE3) revealing the presence of normal sized LDs. Bars = 10 μm. (I) Quantification of supersized LDs in cotyledons derived from mature seeds of the different genotypes. Significant differences between the genotypes were calculated for each genotype using at least 30 images of seeds derived from three plants per genotype (***P < 0.001, Kruskal–Wallis test followed by Dunn's test. Genotypes assigned different lowercase letters are statistically significantly different). (J) Quantification of oil content in seeds reveals a reduction in oil content in the two *ltp6* mutants while the complemented lines have oil levels comparable to the wild‐type Col‐0. Error bars represent standard deviation of seeds from 10 plants/genotype; statistical differences to the wild‐type (WT) were determined for each genotype (*P < 0.05, Kruskal–Wallis test followed by Dunn's test). (K) Germination assay of Col‐0, *ltp6*_45, *ltp6*_A, Arabidopsis *ltp6* lines complemented with AtLTP6 (*ltp6*_45: AtLTP6 #2, *ltp6*_A: AtLTP6 #3), and Arabidopsis *ltp6* mutants transformed with TaLTP6 (*ltp6*_45: TaLTP6 #3, *ltp6*_A: TaLTP6 #5). The germination was performed using surface sterilized and unsterilized Arabidopsis seeds. The *ltp6* mutants and *ltp6* mutants transformed with TaLTP6 show reduced germination rates and the phenomenon is exacerbated in the surface sterilized seeds. Values are averages derived from 1000 seeds per line split into three replicates. Error bars show SD. Statistical differences to the wild‐type were determined for each genotype (*P < 0.05; **P < 0.01, ***P < 0.001; Kruskal–Wallis test, followed by Dunn's test).

Transformation of both Arabidopsis *ltp6* mutant lines with *AtLTP6* under the control of *CaMV35S* promoter (*ltp6*_45: AtLTP6 #2, *ltp6*_A: AtLTP6 #3) restored the normal LD phenotype as might be anticipated (Figure 8D,E), confirming that the loss‐of‐LTP6 function was indeed responsible for the very large LDs in embryo. To test whether TaLTP6 was functionally similar to AtLTP6, we transformed the Arabidopsis *ltp6* mutants with *TaLTP6* also under the control of the *CaMV35S* promoter to see whether mutant phenotypes were reversed (Figure 8). The transformation of the Arabidopsis *ltp6* mutants with *TaLTP6* (*ltp6*_45: TaLTP6 #3, *ltp6*_A: TaLTP6 #5) largely restored the LD phenotype to normal size in embryos, although a few larger sized LDs persisted (Figure 8F,G,I). Visualization of Arabidopsis *ltp6* mutant embryos in stable lines transformed with *TaLTP6* or *AtLTP6* under the strongly active promoter, *CaMV35S* (Benfey et al., 1989; Kiselev et al., 2021) and with *eGFP* appended to the C‐terminus to confirm LTP6 expression, revealed that both proteins were expressed in the transgenics, with GFP fluorescence detected in the embryos and not the untransformed mutants (Figure S13). Interestingly, the quantification of oil in complemented lines revealed that both the TaLTP6‐ and AtLTP6‐containing lines had wild‐type (Col‐0) levels of seed oil content, unlike the mutants that had a significantly reduced oil content (Figure 8J). Overexpression of *AtLTP6* and *TaLTP6* in the Arabidopsis wild‐type background had no influence on LD size as might be expected (Figure 8H) since it was the loss‐of‐function that caused the abnormally large LD phenotypes. Seed oil content in overexpression lines in the Col‐0 background was comparable to that of non‐transformed Col‐0, except for one TaLTP6 overexpression line that showed a small, but significant, increase in seed oil (TaLTP6 OE3; Figure S11). Analysis of FA profiles in seeds of Arabidopsis Col‐0, *ltp6* mutants, complemented *ltp6* mutants and overexpression lines (in the Col‐0 background) did not reveal major differences in composition except that the *ltp6* mutants had a higher C18:3 and C20:2 FA content compared with the wild‐type seeds (Figure S12). The complemented and overexpression lines except TaLTP6 OE3 had higher amounts of C18:1 FAs in comparison with the wild‐type Col‐0 seeds (Figure S12).

**Figure 9:**
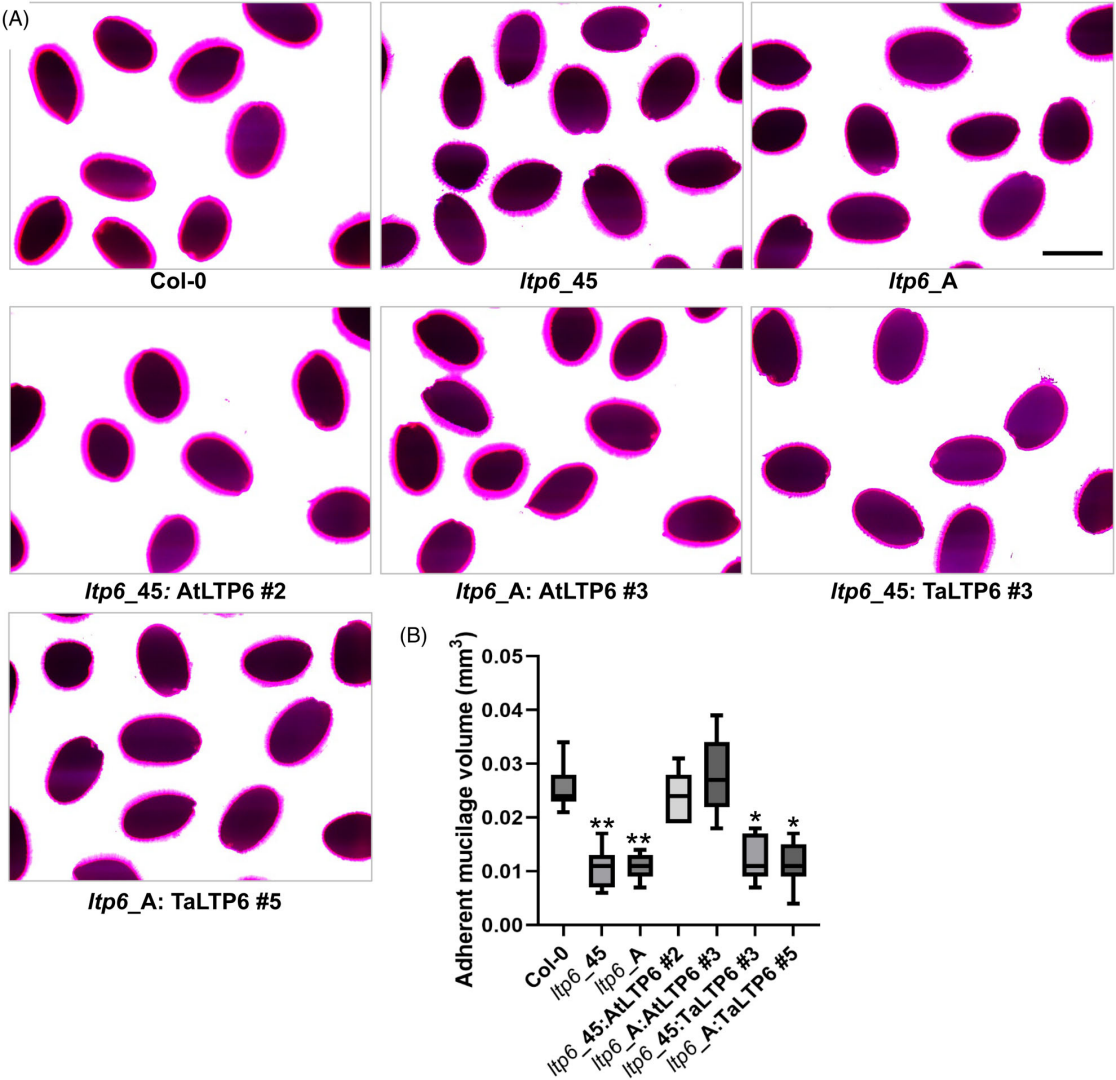
Complementation of Arabidopsis *ltp6* mutants with TaLTP6 does not restore the seed coat phenotype. (A) Adherent mucilage of Col‐0, *ltp6*_45, *ltp6*_A, Arabidopsis *ltp6* lines complemented with AtLTP6 (*ltp6*_45: AtLTP6 #2, *ltp6*_A: AtLTP6 #3), Arabidopsis *ltp6* mutants transformed with TaLTP6 (*ltp6*_45: TaLTP6 #3, *ltp6*_A: TaLTP6 #5) after staining with ruthenium red. Col‐0 and *ltp6* mutant lines complemented with AtLTP6 have a larger mucilage halo compared with the *ltp6* mutants and the mutants transformed with TaLTP6. Images are representative of at least seven images per genotype. Scale bar = 200 μm. (B) The quantification of adherent mucilage volume reveals a smaller mucilage volume in the mutants and the mutants transformed with TaLTP6. Complementation of the mutants with AtLTP6 restores the normal mucilage volume similar to the wild‐type Col‐0. Error bars show SD. Statistical differences to the wild‐type were determined for each genotype (*P < 0.05, **P < 0.01; Kruskal–Wallis test, followed by Dunn's test, *n* ≥ seven seeds).

Analysis of the Arabidopsis seed coat properties of the complemented *ltp6* lines by staining with ruthenium red revealed that AtLTP6 could fully restore the mucilage extrusion phenotypes, similar to wild‐type levels (Figure 9A,B). On the contrary, the Arabidopsis *ltp6* mutant lines complemented with TaLTP6 had a reduced mucilage extrusion phenotype comparable to that observed in the *ltp6* mutant seeds. A seed germination assay revealed that the TaLTP6‐complemented Arabidopsis *ltp6* lines still had a reduced germination rate (Figure 9K). The reduced germination rate was further compounded in the seeds that underwent surface sterilization at levels similar to the situation observed in the *ltp6* mutant background lines. The Arabidopsis *ltp6* lines complemented with the AtLTP6 had germination rates comparable to the wild‐type with and without surface sterilization.

Taken together, our results indicate that LTP6 is essential for efficient storage lipid accumulation and packaging in embryos, and that disruption of lipid storage in *ltp6* loss‐of‐function mutants influences the accumulation of other seed storage components. AtLTP6 also appears to be important for proper seed coat function and the development of correct surface properties upon seed maturity, which is manifested by mucilage defects and poor germination.

## DISCUSSION

Directed genetic manipulation of lipid accumulation in non‐traditional oil seed crops such as pennycress has been aided by foundational work in the model plant Arabidopsis (Guzha et al., 2024; Marks et al., 2021; McGinn et al., 2019). While many of the genes encoding enzymes and proteins involved in storage lipid biosynthesis, modification and compartmentation have been elucidated in Arabidopsis, there still exist gaps in understanding the mechanistic regulation of these processes, and this is especially the case for actual oilseed crops. Here, we identify a protein in pennycress seeds not previously ascribed to seed storage lipid compartmentation before—an isoform of the LIPID TRANSFER PROTEIN gene family—LTP6. Expression of *TaLTP6* in pennycress seeds was positively associated with seed oil content in an unbiased genetic screen (Arias et al., 2023) and was temporally modulated in a manner similar to other genes involved in storage lipid accumulation in developing embryos (Figure 1).

Comparative and functional studies of the TaLTP6 and its close ortholog, AtLTP6, provided compelling evidence that this LTP family member plays a role in storage lipid accumulation and subcellular packaging, although there may be nuanced additional roles for this protein in other tissues of Arabidopsis where this gene is expressed. Transient (Figure 3) and stable (Figure S6) heterologous expression of *TaLTP6* promoted the accumulation of cytoplasmic storage LDs and increased neutral lipid content in leaf cells. In addition, transient expression of GFP‐tagged TaLTP6 revealed an intracellular localization to LDs and ER/LD junctions in *N. benthamiana* leaves (Figure 7; Figure S10), subcellular locations consistent with LTP6 participation in storage lipid packaging. Perhaps most striking was the ability of *TaLTP6* expression in two Arabidopsis *ltp6* loss‐of‐function mutant lines to reverse the marked disruption to LD morphology and storage lipid accumulation to that observed in wild‐type embryos, similar to that of complementation by the native *AtLTP6* (Figure 8).

While the LD, LD/ER localization of TaLTP6 was perhaps surprising, the targeting of LTPs to LDs has been reported previously. For instance, during sunflower seed germination, an LTP (HaAP10) was proposed to shuttle FAs between the LDs and glyoxysomes (Pagnussat et al., 2012). On the contrary, AtLTP6 was localized to the ER (and in epidermal cells, to the apoplast), a phenomenon that has been observed frequently in eukaryotic cells with other LTPs. LTP localization to the ER has been documented, though mostly at contact sites between the ER and other organelles, such as LDs, mitochondria, plastids, and plasma membrane (Gao & Yang, 2018; Khaddaj & Kukulski, 2023; Voeltz et al., 2024). The distinct localization of pennycress and Arabidopsis LTP6 to the LD/ER junctions and the ER, respectively, points to a potential role in intracellular lipid dynamics. Intriguingly, the similarity in pennycress and Arabidopsis LTP6 amino acid sequence, especially at the N‐terminus, did not result in identical localization patterns. In fact, swapping the N‐termini resulted in the chimeric proteins having opposing localization, implicating the N‐terminal region as an important determinant of the differential subcellular localization observed between the two LTP6 orthologs. One possible explanation could be the subtle differences in their predicted protein structures. AtLTP6 has an N‐terminus region forming an α‐helix, predicted to be a signal peptide and projecting perpendicularly to the other four α‐helices, while its C‐terminus does not form any obvious predicted secondary structure (Figure 2C). In contrast, TaLTP6 was predicted to have an N‐terminus lacking definitive α‐helix structure, albeit this unstructured region was predicted with a low confidence score (Figure 2D). The low confidence in the N‐terminus predicted structure derived using Alphafold, especially for TaLTP6 suggests, however, that other mechanisms may be responsible for the differences in localization. It may be that interaction of the N‐termini with other regions in the LTP6 proteins may provide for subtle differences in structure that determine the ultimate subcellular location. Certainly, other explanations exist for the differences in localization between the two orthologues, such as additional sequences in addition to swapped regions and different protein interactors, but this will need to be investigated in more detail in the future.

The pennycress N‐terminus reveals high hydrophobicity (Figures S2E and S4D), which may allow it to interact with the ER. Furthermore, the TaLTP6 localization to LDs suggests that the protein may also be recruited to the LD surface through its presence on the ER, a phenomenon found in class I LD‐associated proteins (Kory et al., 2016; Pan et al., 2025). However, direct targeting to LDs cannot be ruled out because of the distinct localization to LDs observed in Arabidopsis chimeric protein with a TaLTP6 N‐terminus (Figure 7F). The distinct localization of TaLTP6 to ER subdomains (Figure 7A; Figure S10A) also suggests that the protein may be important for LD nucleation by contributing to ER membrane lipid properties that promote lipid condensation and consequently LD formation (Salo, 2023; Thiam & Ikonen, 2021). In eukaryotes, Seipin proteins have been shown to localize to ER/LD junctions and are important for sequestration of TAGs at LD formation sites and their compartmentation into LDs (Cai et al., 2015; Salo, 2023; Schneiter & Choudhary, 2022; Taurino et al., 2018). TaLTP6 may act in a similar manner and may be involved in the accumulation of VLCFA like erucic acid (C22:1) and their efficient incorporation into TAGs which are subsequently stored in LDs. Previous work performed using rice non‐specific LTPs suggests that they can bind VLCFA with a high affinity (Tousheh et al., 2013). The overexpression of Seipins induced a proliferation of cytosolic LDs accompanied by an ER disruption (Cai et al., 2015), and similarly, TaLTP6 overexpression induced the significant accumulation of LDs and neutral lipids. Interestingly, in humans, the oxysterol binding proteins constitute a large LTP family where two members, ORP5 and ORP8, have been shown to control LD biogenesis by recruiting Seipin to mitochondria‐associated ER membrane subdomains (Guyard et al., 2022). It is plausible that TaLTP6 plays a similar role, although further experiments are still needed to evaluate this possibility.

Another possibility is that *TaLTP6* ectopic expression may induce LD proliferation by contributing to the push, pull, and protection of plant lipids (Sagun et al., 2023; Vanhercke et al., 2014). *TaLTP6* may facilitate the efficient recruitment and incorporation of erucic acid to TAGs, hence contributing to the ‘pull’ and compartmentation of erucic acid rich TAGs in pennycress seeds. TaLTP6 localization to LDs suggests that the protein may also help protect the LDs from turnover (Cai & Horn, 2024; Horn et al., 2021; Ischebeck et al., 2020) and, consequently, help explain the contribution of TaLTP6 to the increased LD abundance in the transient assay system. The increased expression of *TaLTP6* identified in the transcriptome‐wide‐associated study (Arias et al., 2023) coincides with stages associated with increased accumulation of erucic acid formation in pennycress embryos (Claver et al., 2024), further supporting the concept that TaLTP6 may be involved in the incorporation of erucic acid into TAGs and subsequent packaging in developing LDs, although this remains to be determined experimentally. Nonetheless, our gene expression data show the highly regulated temporal expression of *TaLTP6*, which is identical to pennycress FAE1 that is responsible for the formation of VLCFA, including erucic acid (Jarvis et al., 2021; Rasoul et al., 2025). We hypothesize that TaLTP6 may function in the intracellular shuttling of VLCFA for more efficient incorporation into TAG biosynthesis and LD formation. The incorporation of VLCFA in TAG biosynthesis has been described as an inefficient process, resulting in reduced abundances of erucic acid in most seed oils (Guan et al., 2014). The increased abundance of *TaLTP6* transcript, and presumably protein, during embryo development may be important for efficient synthesis of TAGs enriched in erucic acid in pennycress seeds. Work performed by other groups, such as Claver et al. (2024) revealed the increased accumulation of erucic acid in the intermediate to latter stages of embryo development, and the highly regulated temporal expression of *TaLTP6* also coincides with increased erucic acid accumulation. Claver et al. (2024) also identified pennycress LTP6 as one of the most abundant lipid‐related transcripts in developing embryos, consistent with our findings. Nonetheless, further experiments will be required to support the hypothesis that LTP6 is involved in VLCFA accumulation. The increased abundance in LDs observed after ectopic expression of chimeric proteins, especially chimeric TaLTP6 targeting ER and possibly LDs, suggests that TaLTP6 may impact LD abundance either directly or indirectly. More experiments are needed to determine the exact role of the protein.

While there are indeed VLCFAs in Arabidopsis seed oil, and AtLTP6 could play an analogous function to that suggested for TaLTP6 in pennycress seeds, other possible explanations exist for the dramatic disruption of LD organization in the Arabidopsis *ltp6* mutant embryos. Because AtLTP6 localizes to the ER, it is plausible that its absence in *ltp6* mutants alters ER membrane composition, resulting in the formation of defective LDs. Lipid and protein properties have been shown to play an integral role in the biogenesis of normal LDs in other eukaryotes (Chung et al., 2019; Dhiman et al., 2020; Klemm et al., 2013). Disruption of proteins involved in maintaining ER phospholipid composition and integrity can result in the formation of aberrant LDs (Fei et al., 2011; Klemm et al., 2013; Olzmann & Carvalho, 2019; Santinho et al., 2020). To probe the potential role of Arabidopsis LTP6 in LD biogenesis, we investigated the protein composition of isolated LDs as well as the microsomal fractions from wild‐type Col‐0 seeds and *ltp6* mutants. Interestingly, there was an increased abundance of microsomal associated proteins in the LD fraction as well as LD‐associated proteins in the microsomal fraction of the mutants (Figure 5D–I; Figure S6C) suggesting that the LDs remain entangled in the ER matrix. Another possibility is that the LD monolayer derived from the ER has compromised integrity resulting in the fusion of LDs as more are packed in the seed cells during embryo development. This may explain why the fused LD phenotype becomes more prominent as the embryos reach maturity. Arabidopsis LTP6 may be important for LD integrity, especially during late embryo development stages when rapid moisture loss occurs during desiccation. According to public databases (https://www.bar.utoronto.ca/efp//cgi-bin/efpWeb.cgi?dataSource=Abiotic_Stress&mode=Absolute&primaryGene=At3g08770&secondaryGene=At3g27340&override=&threshold=3909.38&modeMask_low=None&modeMask_stddev=None) the expression of *LTP6* is induced upon moisture stress suggesting a potential role in adaptation to moisture stress, especially during late embryo development stages. Evidence for this possibility could be the increased abundance of storage proteins in the seeds of Arabidopsis *ltp6* mutants and the concomitant, significant reduction in carbohydrates and oil (Figure 4G–I). The increased production of seed proteins upon various abiotic stresses has been reported in other studies (De Souza et al., 2015; Kashyap et al., 2024; Tanaka et al., 2021). Follow‐up experiments to determine possible interactions with lipid‐related proteins or LD biogenesis factors are needed to determine the exact role of LTP6 in LD biogenesis or stability.

Interestingly, the seeds derived from Arabidopsis *ltp6* mutants showed reduced or delayed germination and the seedling growth was less vigorous (Figure 6; Figure S7). This may be due to a defect in lipid mobilization, which is critical for germination and seedling growth. Analysis of BODIPY‐stained seeds that fail to germinate reveals the presence of uncompartmentalized lipids (Figure 6D), which may impair the efficient lipolysis and β‐oxidation of stored lipids, processes important for germination and early seedling growth. Similar germination and growth defects have been previously reported in other PR14 family mutants, like *ltp3*, which also exhibited compromised lipid mobilization (Pagnussat et al., 2015). We, however, cannot rule out possible additional effects of AtLTP6 disruption on seed coat properties of the mutant seeds, as evidenced by the reduced volume of adherent mucilage in the mutant seeds (Figure S9). It is possible that there are surface lipid‐related functions for the AtLTP6, especially given its expression in seed coat tissues. Arabidopsis LTP2, a member of the PR‐14, influences extracellular lipid deposition and cuticle organization, which can indirectly affect the integrity of pectin‐rich cell walls by altering hydration dynamics and mechanical properties at the cell wall–cuticle interface (Jacq et al., 2017). Further experiments are, however, still needed to ascertain the validity of this assertion.

Functional assays of Arabidopsis and *TaLTP6*, performed by complementing the Arabidopsis *ltp6* mutants with *TaLTP6*, revealed partial restoration of LD morphology in embryos. While some supersized LDs persisted, their occurrence and size were significantly reduced compared with the mutants, and normal seed oil content was restored (Figure 8F,G,I,J), suggesting that AtLTP6 and TaLTP6 proteins share similar functions in embryos, although not perhaps exact overlap. On the contrary, the transformation of Arabidopsis *ltp6* mutants with *TaLTP6* did not rescue the germination phenotype or seed coat properties (Figures 8K and 9). Perhaps TaLTP6 evolved a more specialized role in the formation and storage of TAGs enriched in VLCFAs like erucic acid, while losing functions related to seed coat integrity. This concept is supported by the lack of endogenous *TaLTP6* expression in pennycress seed coat tissues. We propose that the two protein homologs share functions and are important for LD biogenesis as well as LD integrity. However, more experiments will be needed to determine the nature of the differences in functions between the two isoforms, particularly in seed coats and perhaps the surfaces of other plant tissues.

## EXPERIMENTAL PROCEDURES

### Plant material, growth conditions, and transformations

Arabidopsis plants were grown in soil or on plates containing half‐strength Murashige and Skoog media (Murashige & Skoog, 1962) and supplemented with hygromycin for selection of complemented or overexpression lines and grown in a growth chamber at 22°C with a 16‐h‐day/8‐h‐night cycle and 50 μE m^−2^ sec^−1^ light intensity. Col‐0 and transgenic Arabidopsis plants were grown together at the same time to harvest seed for further studies. All Arabidopsis experiments were conducted in the Col‐0 ecotype background. Arabidopsis stable or complemented lines plants were generated using the floral dip method (Clough & Bent, 1998) with the *Agrobacterium tumefaciens* strain GV3101 containing the binary plasmid pMDC32/At/TaLTP6 or pMDC84/At/TaLTP6:eGFP. PCR was used to detect the presence of the transgene. Pennycress was grown on soil using four‐inch pots and at a density of four plants per pot. The plants were grown in a greenhouse under a controlled environment with a light cycle of 16 h light/8 h dark, fluorescent lighting, 175–250 μE m^−2^ sec^−1^ light intensity and at a temperature of 22°C.

*Nicotiana benthamiana* plants for use in transient expression experiments were grown on soil at 28°C under a 14/10‐h light/dark cycle in a growth chamber set at 50 μE m^−2^ sec^−1^ light intensity. Well‐developed leaves of 4‐week‐old *N. benthamiana* plants were infiltrated with *A. tumefaciens* strain GV3101, carrying appropriate binary vectors. Detailed procedures for *A. tumefaciens* growth, transformation, infiltration, and processing of *N. benthamiana* leaf material for microscopy are described in Cai et al. (2015).

### Germination assays

Arabidopsis seeds were plated on one‐half‐strength MS 0.8% agar media or on soil and incubated at 4°C for 2 days in the dark. Seeds sterilization was performed by soaking the seeds in 12.5% bleach for 15 min before rinsing at least three times with sterile milliQH_2_O. Plates and/or pots were then placed in a growth chamber set at 22°C with a 16‐h day/8‐h night cycle and 50 μE m^−2^ sec^−1^ light intensity. The number of seeds that germinated was recorded on a daily basis.

### RNA seq data analysis

Raw counts were transformed using DeSeq2 v1.30.1 (Love et al., 2014) to perform PCA with PCAGO (Gerst & Hölzer, 2019). Raw RNA seq data were pre‐processed as described by Johnston et al. (2022, 2024) and is available on the National Center for Biotechnology Information (BioProject Accession: PRJNA808106); pre‐processed reads were mapped to the pennycress reference from Dorn et al. (2015), resulting in >95% mapping. *Arabidopsis thaliana* homologs were assigned to pennycress transcript sequences for annotation as previously described (Garcia Navarrete et al., 2022; Johnston et al., 2022; Krishnakumar et al., 2015; Pasha et al., 2020).

### Plasmid construction

The reagents used for construction of plasmids were purchased from New England Biolabs (Ipswich, MA, USA, https://www.neb.com/), Thermo Fisher Scientific (Rockford, IL, USA, https://www.thermofisher.com/) or Invitrogen (Carlsbad, CA, USA, http://www.invitrogen.com/), and oligonucleotides were synthesized by Genwiz (South Plainfield, NJ, USA, http://www.genwiz.com/). Plasmids used for expression in plants were driven by the Cauliflower Mosaic Virus 35S (CaMV35S) promoter. Full‐length coding DNA sequences of Arabidopsis and pennycress LTP6 were amplified from embryo cDNA using primers listed in Table S1. The PCR products and the plant expression binary vector plasmids pMDC32 and pMDC84 (with C‐terminus GFP) were digested with restriction enzymes PacI and AscI. Cloning was performed as described in Guzha et al. (2024).

### Confocal microscopy

Confocal laser scanning microscopy (CLSM) images were acquired *in situ* in *N. benthamiana* leaves, Arabidopsis leaves, embryos excised from Arabidopsis seeds, and isolated LDs using a Zeiss LSM 710 confocal laser scanning microscope retrofitted with an Airyscan enhanced‐imaging head. Processing of acquired images was done using Zeiss Zen software (Black edition and Blue edition; v. 2011). Infiltrated leaves (3 or 4 days post infiltration) of 4‐weeks old *N. benthamiana* or leaves of Arabidopsis stable expression lines were collected using a hole punch and stained with either BODIPY 493/503 or Nile red (Sigma‐Aldrich, St. Louis, MO, USA) neutral lipid stains at a concentration of 4 μg ml^−1^ in 50 mM PIPES buffer, as described in Guzha et al. (2024). Whole Arabidopsis embryos were collected after soaking the seeds in water for 15 min to enable removal of seed coats. For pennycress the seeds were soaked in water for 30 min to facilitate seed coat removal. The embryonic axis was excised using a razor blade and sections were made on both embryonic axis and cotyledons using a razor blade before staining with BODIPY. GFP (and BODIPY), Nile red and chloroplast autofluorescence were excited by a 488‐nm laser and collected in a spectrum of 500–540, 560–620, and 640–720 nm, respectively. CFP (for the ER marker ER marker protein Kar2‐CFP‐HDEL used in *N. benthamiana* transient assays) was excited by a 405 laser and the emission signal was collected in a spectrum of 450–490 nm.

### LD isolation

Approximately 200 mg of Arabidopsis seeds per plant and using three plants per genotype was used to obtain LD fractions, microsomes and total cellular fractions. LD‐enriched fractions were obtained as described previously (Horn et al., 2021) and stained with BODIPY for CLSM. Analysis of proteins enriched in LDs or ER fractions was performed as described in Horn et al. (2021). Microsomal proteins in LD fraction were quantified after normalization of the relative iBAQ values as described in Niemeyer et al. (2022).

### Biomass extraction and quantification

Biomass components (oil, protein and/or starch) were extracted from *N. benthamiana* leaves or Arabidopsis seeds as described previously (Cocuron et al., 2014; Guzha et al., 2024; Johnston et al., 2022).

### Mucilage staining with ruthenium red

Mucilage staining was performed as described in Guzha et al. (2022) and Scholz et al. (2023), with slight modifications. Five milligrams of Arabidopsis seeds were placed in 500 μl milliQH_2_O in an Eppendorf tube and then shaken gently at room temperature for 1 h using a rotary shaker. Water was gently removed before 500 μl of 0.02% ruthenium red (Sigma‐Aldrich) was added followed by another gentle shaking for 15 min. The ruthenium red solution was removed, and seeds were again resuspended in ultrapure milliQ H_2_O. A droplet with stained seeds was placed on a microscopic slide and viewed under a light microscope for imaging. The shape of the seed was taken as a spheroid as described in Guzha et al. (2022). The volume of the adherent mucilage was calculated by subtracting the volume of the seed alone from the total volume of the seed with mucilage using the formula: volume = 4/3 × 1/8 × length × width^2^ as described in Guzha et al. (2022).

## AUTHOR CONTRIBUTIONS

AG, APA, and KDC designed the research. AG, JVS, PW, and CJ, performed the experiments and analyzed the data. APA and KDC supervised the research. AG and KDC drafted the manuscript with contributions, edits, and approval from all other authors.

## CONFLICT OF INTEREST

None declared.

## Supporting information


**Figure S1.** (A) Graph showing the abundance of neutral lipids in pennycress embryos from two accessions (high oil and low oil) at maturity.
**Figure S2.** (A) Polypeptide sequence alignment of TaLTP6 and Arabidopsis PR14 family proteins. Identical amino acid residues found in the polypeptide sequences are highlighted red and blue or green and indicated with asterisks and colons or periods, respectively. Note the conserved eight‐cysteine residues forming disulfide bridges and a putative signal peptide in the N‐terminus. Predicted signal peptides in Arabidopsis (B) and pennycress (C) polypeptide sequences. Hydropathy plots derived from amino acid sequence of AtLTP6 (D) and TaLTP6 (E) based on the Kyte–Doolittle scale. A hydrophobic region is present in the N‐terminus on both sequences.
**Figure S3.** eFP browser (https://bar.utoronto.ca/efp_arabidopsis/cgi‐bin/efpWeb.cgi) data revealing the relative expression of *AtLTP6* in different organs (A), in developing embryos (B) during seed development (C).
**Figure S4.** Prediction of transmembrane‐spanning domains in Arabidopsis (A) and pennycress (B) LTP6. The TMHMM algorithm available at http://www.cbs.dtu.dk/services/TMHMM/ was used to analyze AtLTP6 and TaLTP6 deduced polypeptide sequences for the presence of putative hydrophobic transmembrane‐spanning sequences. The graphs show an obvious absence of such sequences in AtLTP6 and TaLTP6. Helical wheel projection of amino acid residues 4–21 in AtLTP6 (C) and TaLTP6 (D), respectively. Hydrophobic amino acid residues are colored yellow. The direction of the arrowhead in the helical wheel indicates the position of the hydrophobic face along this region.
**Figure S5.** Three‐D homology models of Arabidopsis and pennycress LTP6, revealing the typical four‐α‐helix‐bundle architecture comprising the hydrophobic cavity in the protein core.
**Figure S6.** Stable overexpression of TaLTP6 coding sequences is associated with increased LD abundance in *A. thaliana* leaves.
**Figure S7.** Disruption of AtLTP6 in Arabidopsis seeds results in reduced germination rates.
**Figure S8.** Morphological phenotypes of Col‐0, *ltp6_*45, and *ltp6_*A grown under the same conditions at 6 weeks after germination (A) and at maturity (B). Average plant height at maturity (C), plant dry weight (D) and total dry seed weight per plant (E) for the three genotypes.
**Figure S9.** Arabidopsis *ltp6* mutants have disrupted seed coat properties.
**Figure S10.** Representative CLSM images (*z*‐sections) of *N. benthamiana* leaf mesophyll cells co‐transformed with TaLTP6:eGFP (A) with CFP‐HDEL serving as the ER marker protein and LDs are stained red with Nile red while blue is autofluorescence from the chloroplasts. TaLTP6:eGFP localizes to LDs and LD/ER junctions (arrow). Image is a zoom in on Figure 9A. (B) Representative CLSM images (*z*‐sections) of *A. thaliana* leaf cells stably overexpressing pennycress LTP6 with GFP appended to the C‐terminus reveals a localization to LDs or in close proximity to LDs. LDs are stained red with Nile red. Bars = 20 μm. (C) eFP browser (https://bar.utoronto.ca/efp_arabidopsis/cgi‐bin/efpWeb.cgi) data revealing the predicted subcellular localization of ATLTP6. (D) Polypeptide sequence alignment of TaLTP6 and AtLTP6. Underlined area represents regions swapped in the N‐terminus swap experiment and asterisk show conserved cysteine residues.
**Figure S11.** Quantification of oil content in seeds reveals a reduction in oil content in the two *ltp6* mutants while the stable Arabidopsis AtLTP6 overexpression lines (AtLTP6 OE2 and AtLTP6 OE3) have oil levels similar to the wild‐type Col‐0.
**Figure S12.** Variation in the fatty acid profiles in Arabidopsis seeds derived from Col‐0, _45, _A, Arabidopsis *ltp6* lines complemented with AtLTP6 (*ltp6*_45: AtLTP6 #2, *ltp6*_A: AtLTP6 #3), Arabidopsis *ltp6* mutants transformed with TaLTP6 (*ltp6*_45: TaLTP6 #3, *ltp6*_A: TaLTP6 #5), Arabidopsis AtLTP6 overexpression lines (AtLTP6 OE2, AtLTP6 OE3) and Arabidopsis lines overexpressing TaLTP6 (TaLTP6 OE3 and TaLTP6 OE5).
**Figure S13.** Representative Arabidopsis *ltp6* lines complemented with TaLTP6 (A), AtLTP6 (B) and untransformed *ltp6*_45 (C) revealing expression of the transgene in the developing embryos of the mutants. As expected, the untransformed mutant does not show any eGFP fluorescence (C). Bars = 10 μm.

**Table S1.** List of primers.

**Table S2.** Proteins identified in wild‐type Col‐0 microsome/membrane fraction.

**Dataset S1.** Volcano plots data.

## Data Availability

All relevant data can be found within the manuscript and its Supporting Information.
