# Supplementary material for: Functional analysis of LIPID TRANSFER PROTEIN 6 (LTP6) in pennycress and Arabidopsis reveals divergent roles in oil storage and seed coat development
Source: Plant J. 2026 Jul 13;127(1):e71038. doi: 10.1111/tpj.71038 (PMC13363014; doi:10.1111/tpj.71038)
Supplement: Supplementary file 1 — Figure S1. (A) Graph showing the abundance of neutral lipids in pennycress embryos from two accessions (high oil and low oil) at maturity. Figure S2. (A) Polypeptide sequence alignment of TaLTP6 and Arabidopsis PR14 family proteins. Identical amino acid residues found in the polypeptide sequences are highlighted red and blue or green and indicated with asterisks and colons or periods, respectively. Note the conserved eight‐cysteine residues forming disulfide bridges and a putative signal peptide in the N‐terminus. Predicted signal peptides in Arabidopsis (B) and pennycress (C) polypeptide sequences. Hydropathy plots derived from amino acid sequence of AtLTP6 (D) and TaLTP6 (E) based on the Kyte–Doolittle scale. A hydrophobic region is present in the N‐terminus on both sequences. Figure S3. eFP browser (https://bar.utoronto.ca/efp_arabidopsis/cgi‐bin/efpWeb.cgi) data revealing the relative expression of AtLTP6 in different organs (A), in developing embryos (B) during seed development (C). Figure S4. Prediction of transmembrane‐spanning domains in Arabidopsis (A) and pennycress (B) LTP6. The TMHMM algorithm available at http://www.cbs.dtu.dk/services/TMHMM/ was used to analyze AtLTP6 and TaLTP6 deduced polypeptide sequences for the presence of putative hydrophobic transmembrane‐spanning sequences. The graphs show an obvious absence of such sequences in AtLTP6 and TaLTP6. Helical wheel projection of amino acid residues 4–21 in AtLTP6 (C) and TaLTP6 (D), respectively. Hydrophobic amino acid residues are colored yellow. The direction of the arrowhead in the helical wheel indicates the position of the hydrophobic face along this region. Figure S5. Three‐D homology models of Arabidopsis and pennycress LTP6, revealing the typical four‐α‐helix‐bundle architecture comprising the hydrophobic cavity in the protein core. Figure S6. Stable overexpression of TaLTP6 coding sequences is associated with increased LD abundance in A. thaliana leaves. Figure S7. Disruption of AtLTP6 in [file TPJ-127-0-s003.pptx]

## Slide 1
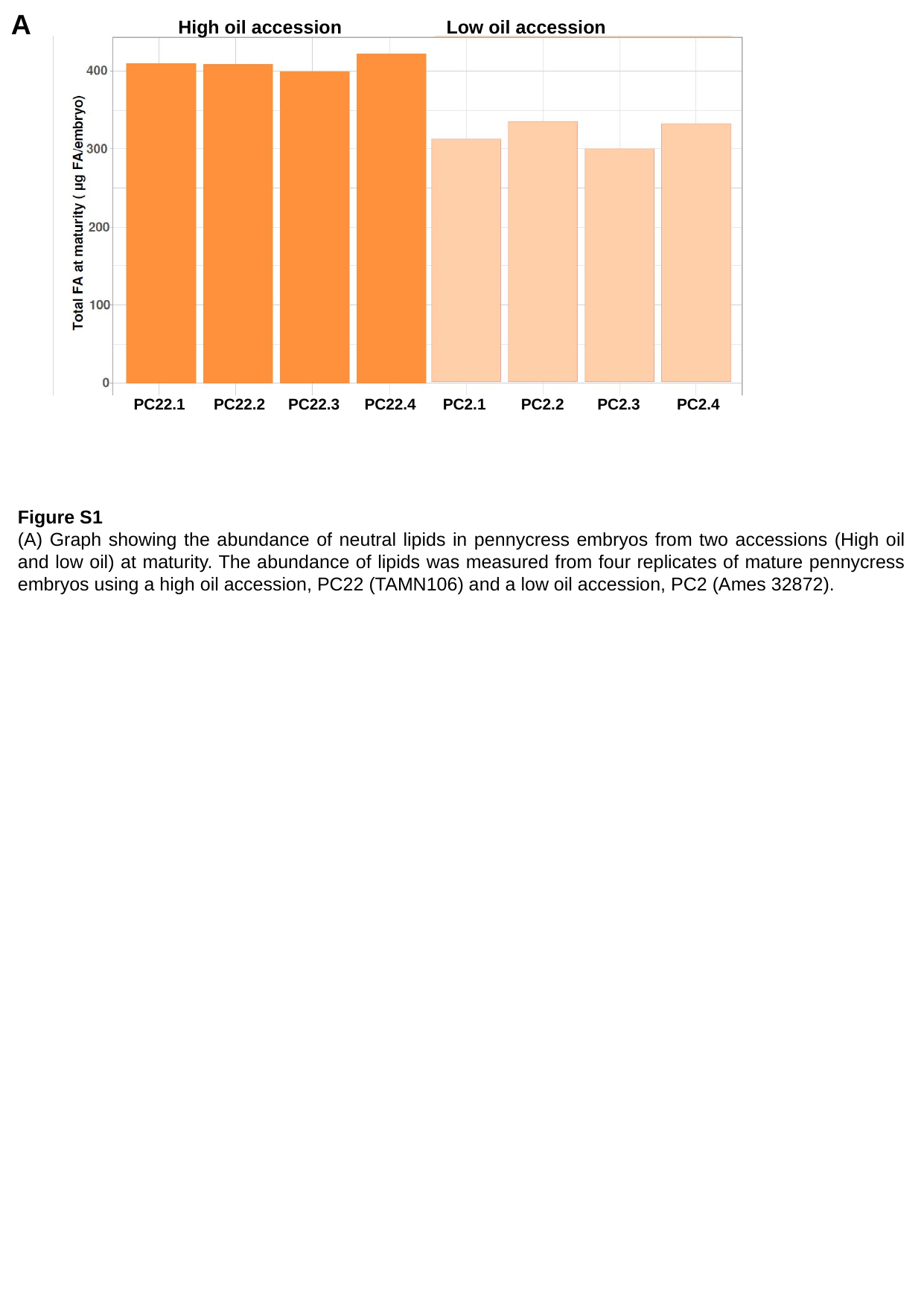

A
High oil accession
Low oil accession
PC22.2
PC22.3
PC22.4
PC2.1
PC2.2
PC2.3
PC2.4
PC22.1
Figure S1
(A) Graph showing the abundance of neutral lipids in pennycress embryos from two accessions (High oil and low oil) at maturity. The abundance of lipids was measured from four replicates of mature pennycress embryos using a high oil accession, PC22 (TAMN106) and a low oil accession, PC2 (Ames 32872).

## Slide 2
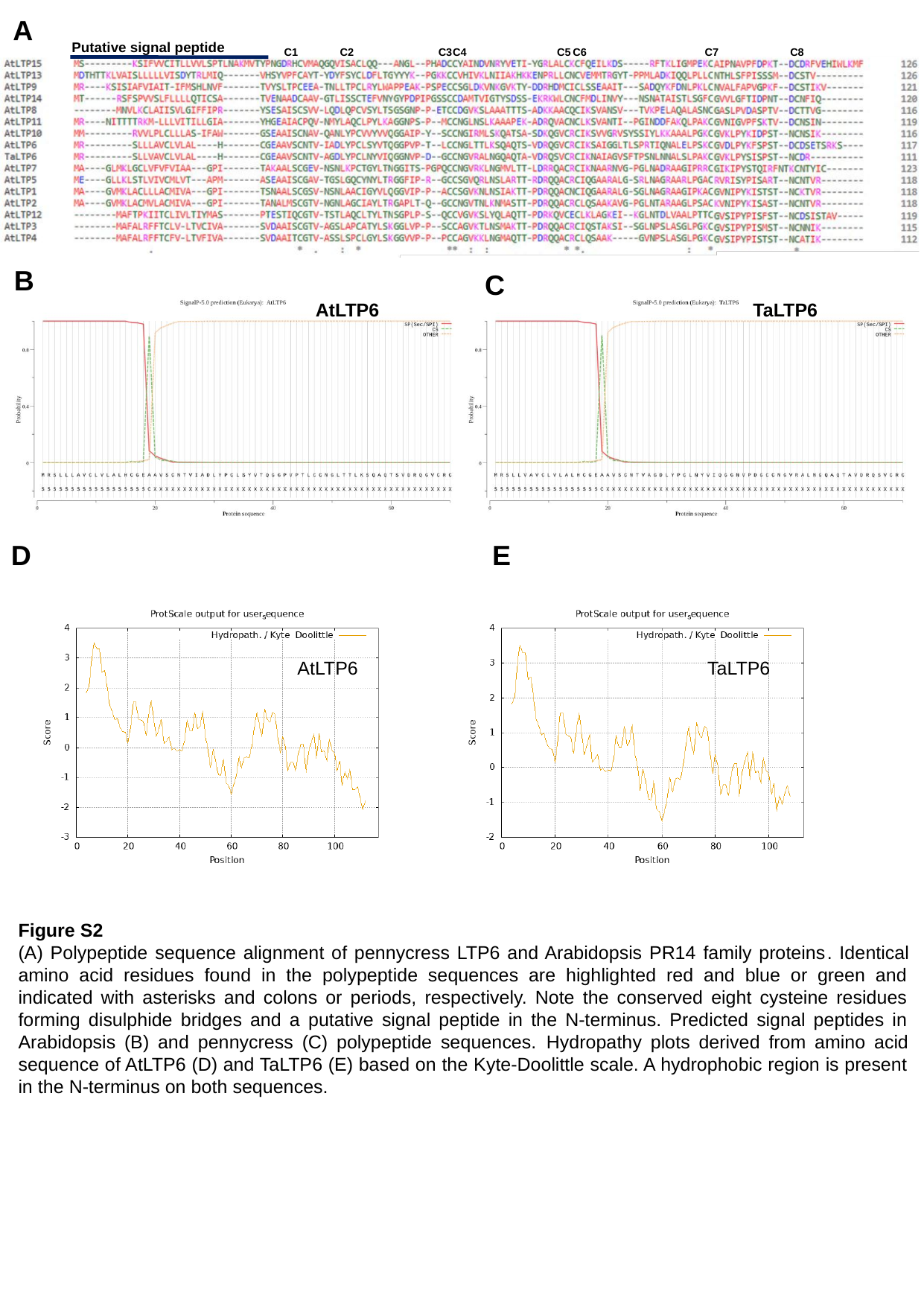

A
Putative signal peptide
C1
C2
C3
C4
C5
C6
C7
C8
B
C
AtLTP6
TaLTP6
D
E
AtLTP6
TaLTP6
Figure S2
(A) Polypeptide sequence alignment of pennycress LTP6 and Arabidopsis PR14 family proteins. Identical amino acid residues found in the polypeptide sequences are highlighted red and blue or green and indicated with asterisks and colons or periods, respectively. Note the conserved eight cysteine residues forming disulphide bridges and a putative signal peptide in the N-terminus. Predicted signal peptides in Arabidopsis (B) and pennycress (C) polypeptide sequences. Hydropathy plots derived from amino acid sequence of AtLTP6 (D) and TaLTP6 (E) based on the Kyte‐Doolittle scale. A hydrophobic region is present in the N‐terminus on both sequences.

## Slide 3
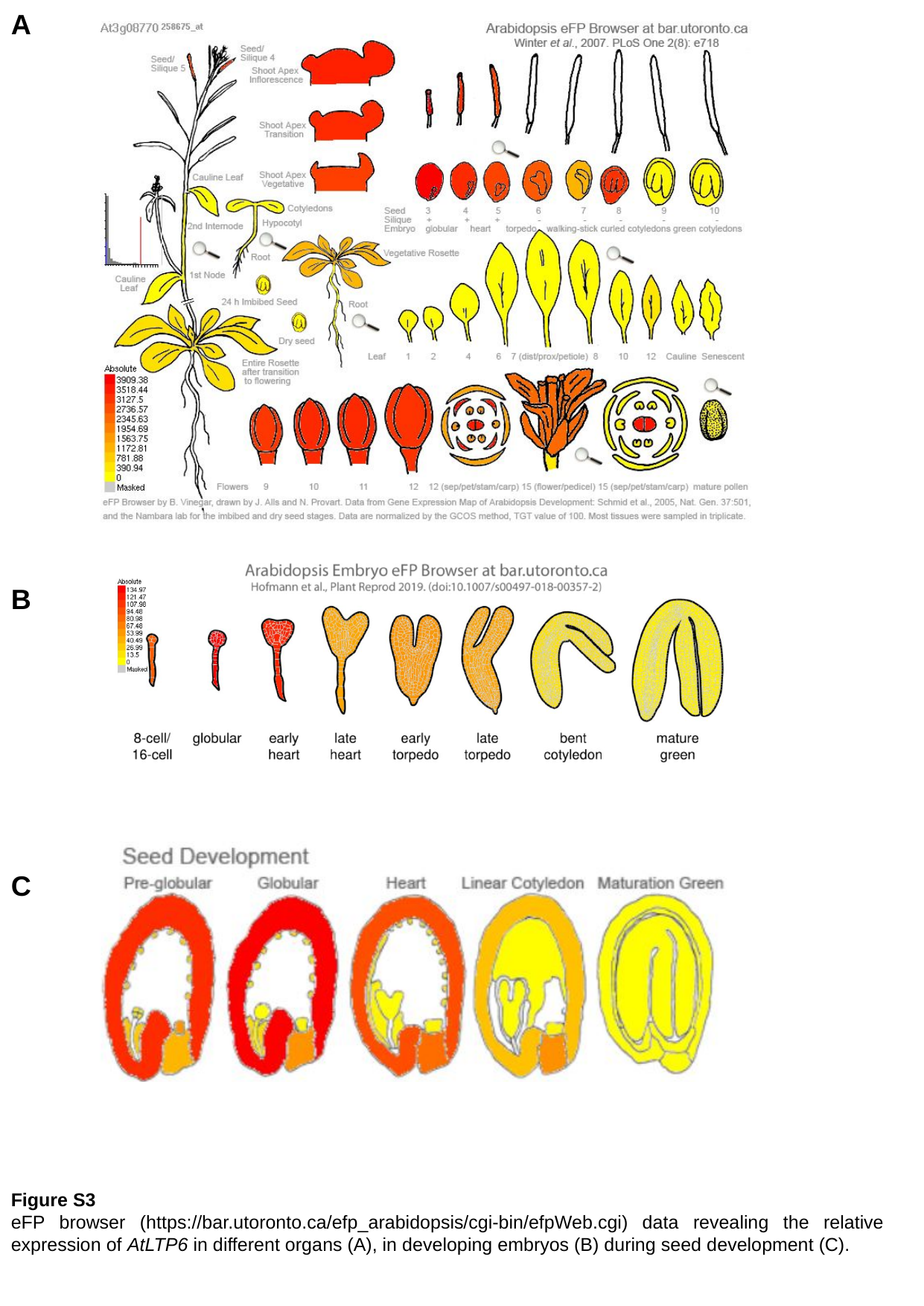

A
B
C
Figure S3
eFP browser (https://bar.utoronto.ca/efp_arabidopsis/cgi-bin/efpWeb.cgi) data revealing the relative expression of AtLTP6 in different organs (A), in developing embryos (B) during seed development (C).

## Slide 4
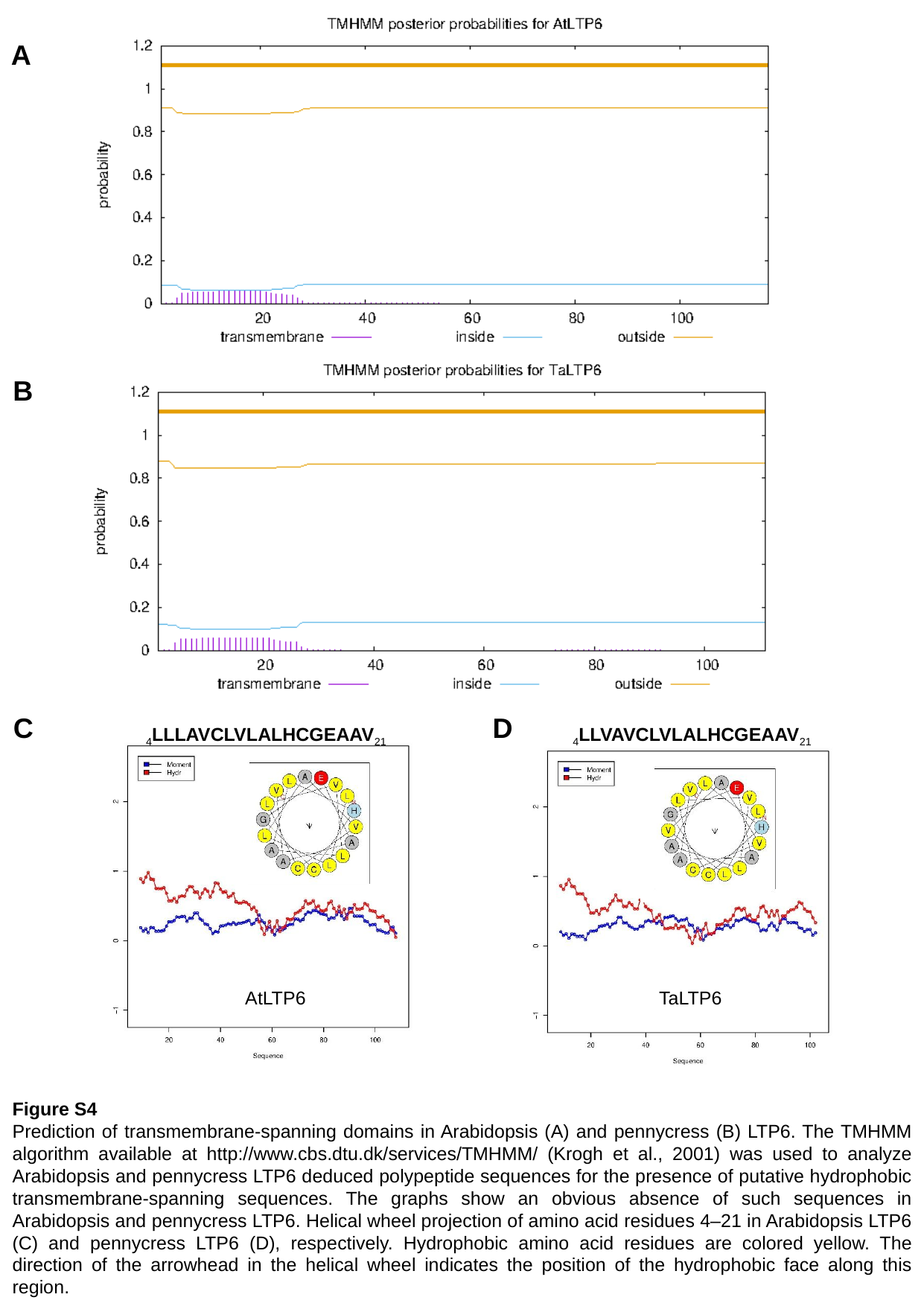

A
B
D
C
4LLLAVCLVLALHCGEAAV21
4LLVAVCLVLALHCGEAAV21
AtLTP6
TaLTP6
Figure S4
Prediction of transmembrane-spanning domains in Arabidopsis (A) and pennycress (B) LTP6. The TMHMM algorithm available at http://www.cbs.dtu.dk/services/TMHMM/ (Krogh et al., 2001) was used to analyze Arabidopsis and pennycress LTP6 deduced polypeptide sequences for the presence of putative hydrophobic transmembrane-spanning sequences. The graphs show an obvious absence of such sequences in Arabidopsis and pennycress LTP6. Helical wheel projection of amino acid residues 4–21 in Arabidopsis LTP6 (C) and pennycress LTP6 (D), respectively. Hydrophobic amino acid residues are colored yellow. The direction of the arrowhead in the helical wheel indicates the position of the hydrophobic face along this region.

## Slide 5
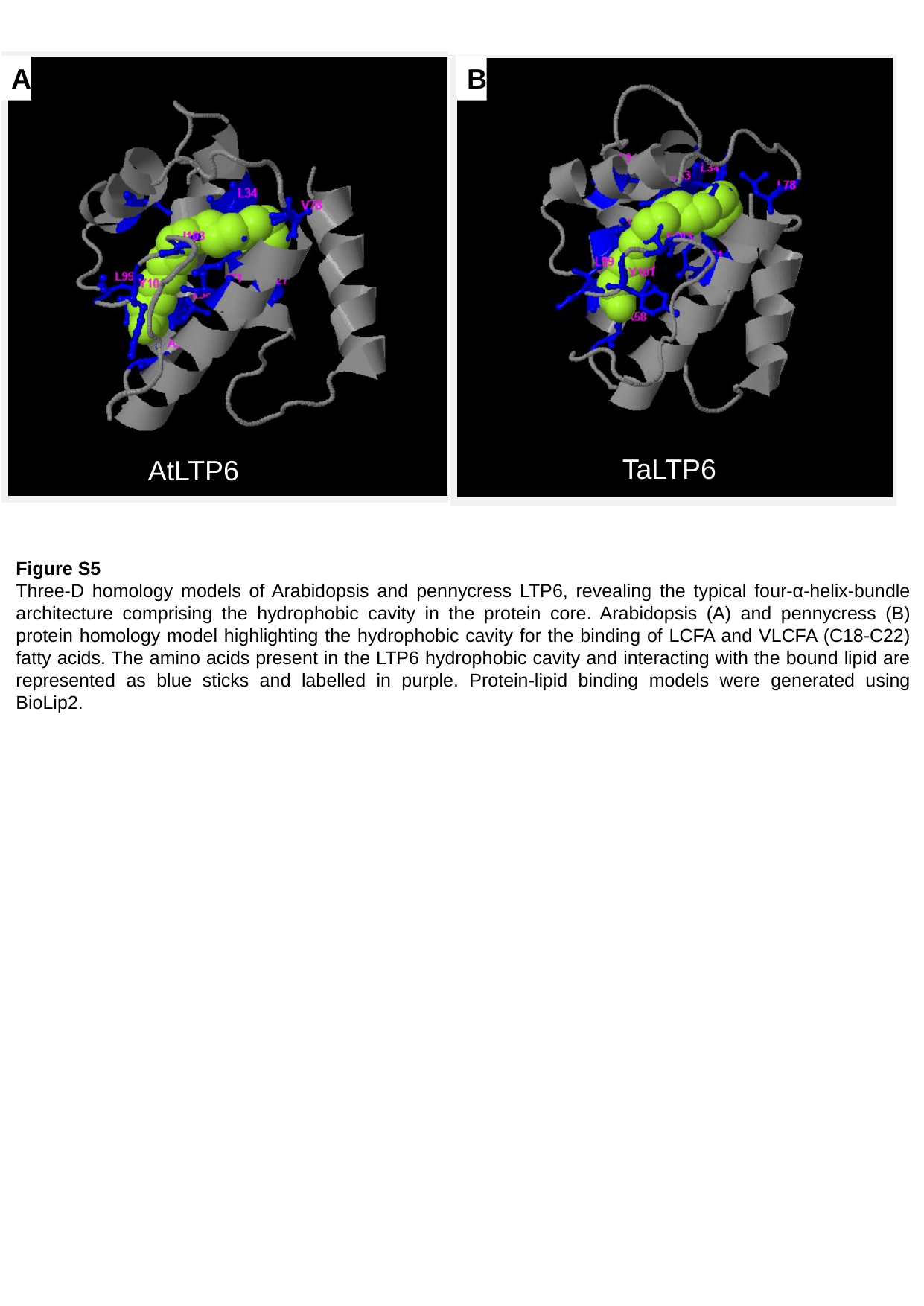

A
B
TaLTP6
AtLTP6
Figure S5
Three-D homology models of Arabidopsis and pennycress LTP6, revealing the typical four-α-helix-bundle architecture comprising the hydrophobic cavity in the protein core. Arabidopsis (A) and pennycress (B) protein homology model highlighting the hydrophobic cavity for the binding of LCFA and VLCFA (C18-C22) fatty acids. The amino acids present in the LTP6 hydrophobic cavity and interacting with the bound lipid are represented as blue sticks and labelled in purple. Protein-lipid binding models were generated using BioLip2.

## Slide 6
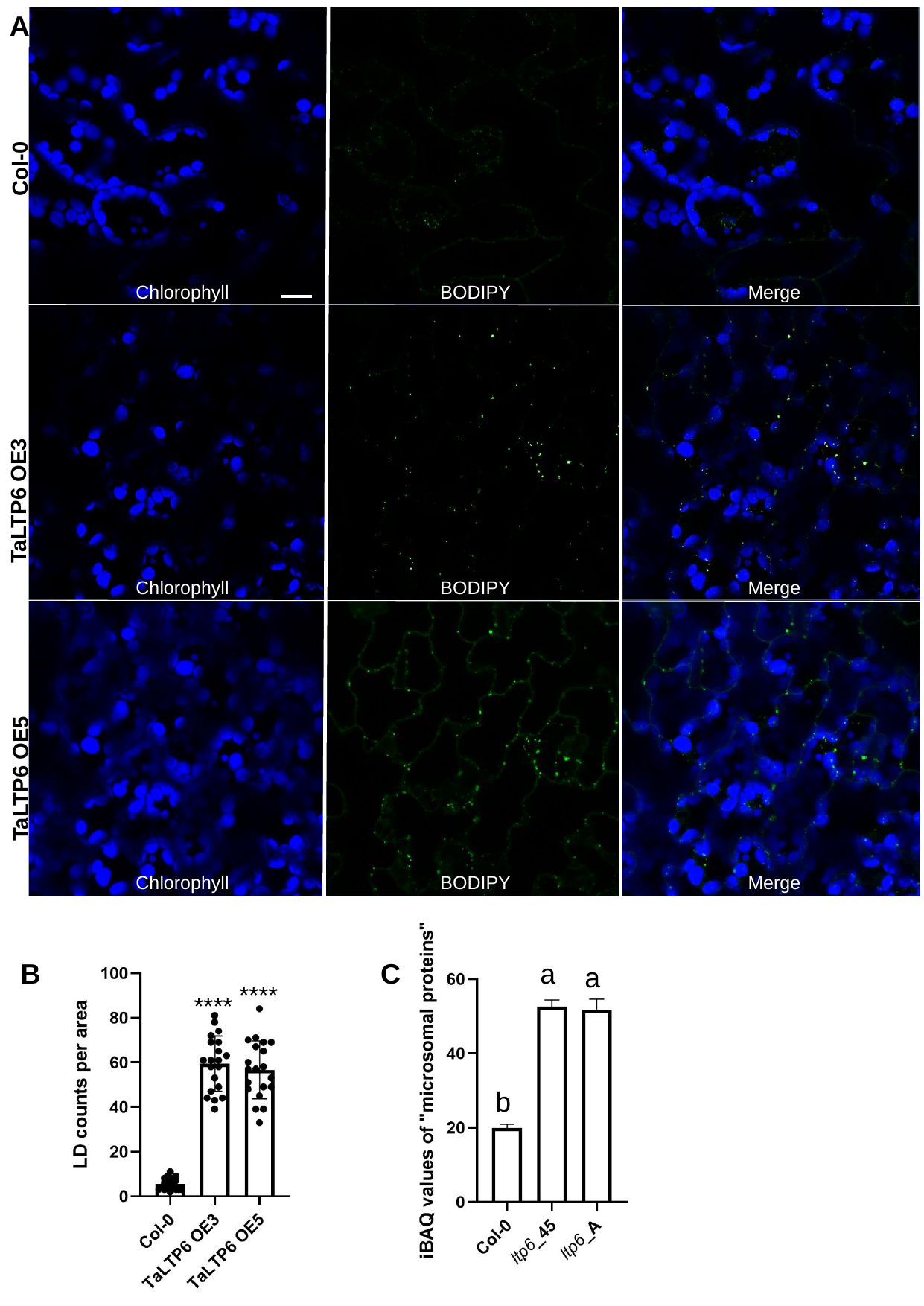

A
Col-0
Merge
BODIPY
Chlorophyll
TaLTP6 OE3
Merge
BODIPY
Chlorophyll
TaLTP6 OE5
Merge
BODIPY
Chlorophyll
B
C
a
a
****
****
b

## Slide 7
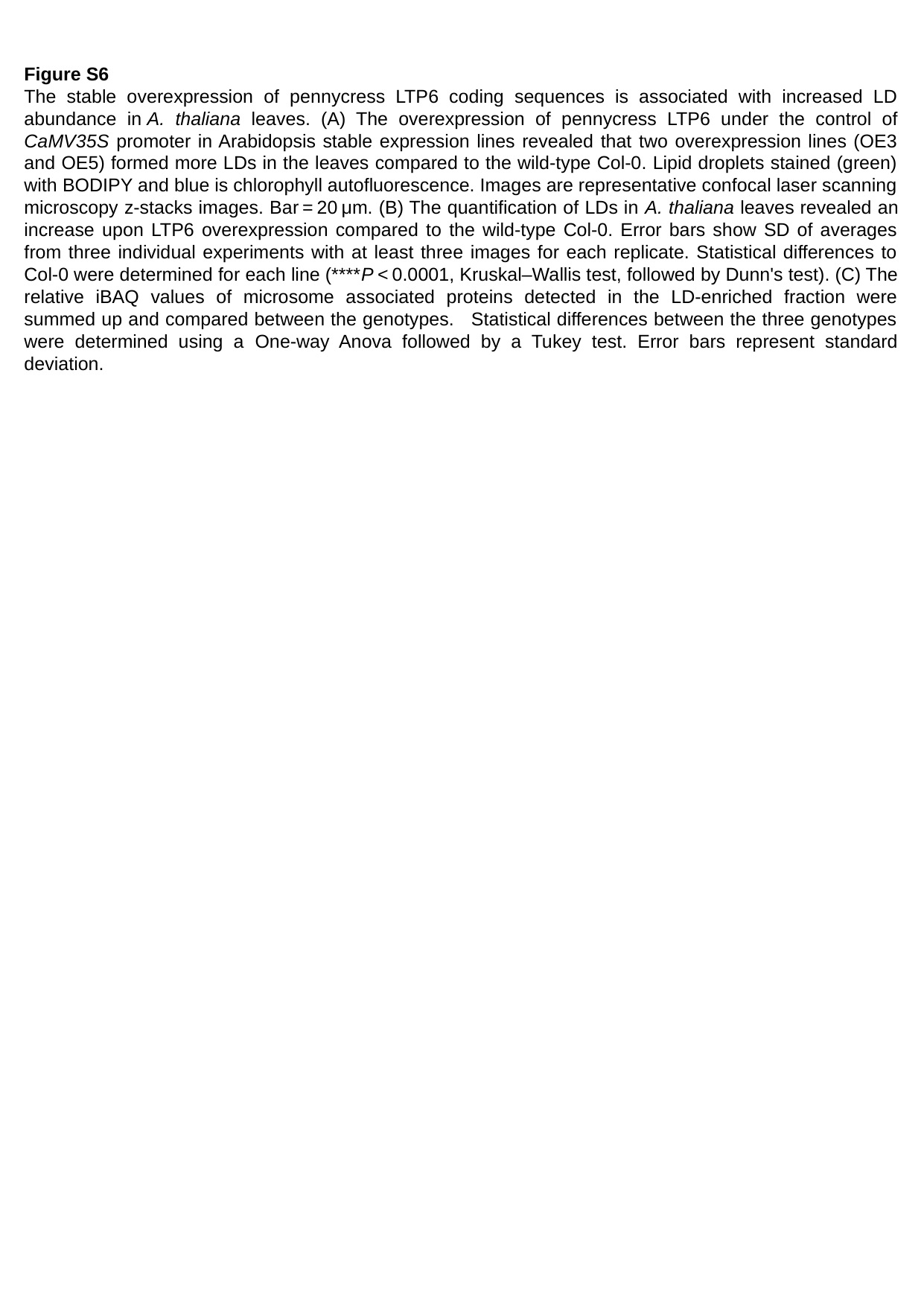

Figure S6
The stable overexpression of pennycress LTP6 coding sequences is associated with increased LD abundance in A. thaliana leaves. (A) The overexpression of pennycress LTP6 under the control of CaMV35S promoter in Arabidopsis stable expression lines revealed that two overexpression lines (OE3 and OE5) formed more LDs in the leaves compared to the wild-type Col-0. Lipid droplets stained (green) with BODIPY and blue is chlorophyll autofluorescence. Images are representative confocal laser scanning microscopy z-stacks images. Bar = 20 μm. (B) The quantification of LDs in A. thaliana leaves revealed an increase upon LTP6 overexpression compared to the wild-type Col-0. Error bars show SD of averages from three individual experiments with at least three images for each replicate. Statistical differences to Col-0 were determined for each line (****P < 0.0001, Kruskal–Wallis test, followed by Dunn's test). (C) The relative iBAQ values of microsome associated proteins detected in the LD-enriched fraction were summed up and compared between the genotypes.   Statistical differences between the three genotypes were determined using a One-way Anova followed by a Tukey test. Error bars represent standard deviation.

## Slide 8
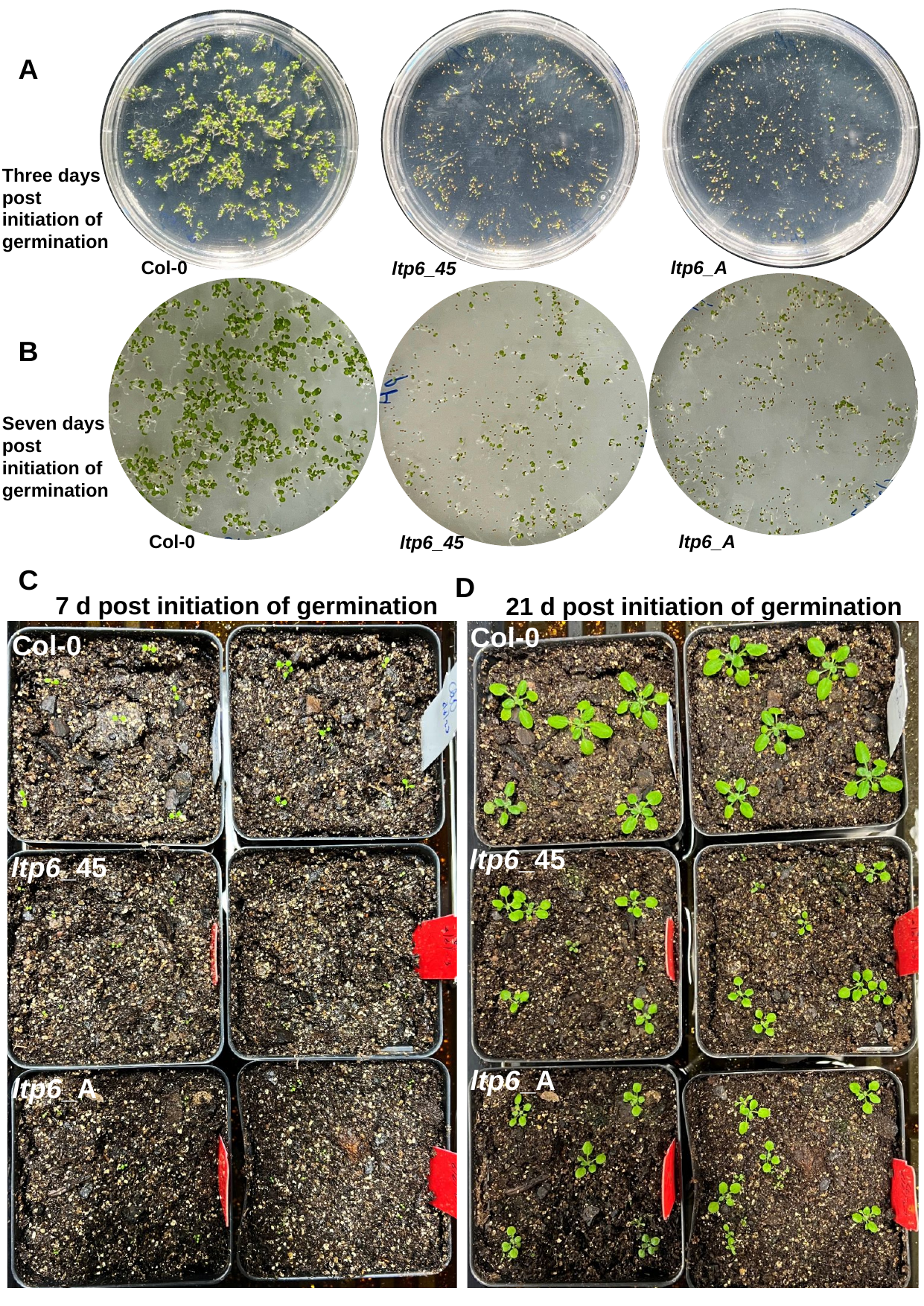

A
Three days post initiation of germination
Col-0
ltp6_A
ltp6_45
B
Seven days post initiation of germination
Col-0
ltp6_A
ltp6_45
C
D
7 d post initiation of germination
21 d post initiation of germination
Col-0
Col-0
ltp6_45
ltp6_45
ltp6_A
ltp6_A

## Slide 9
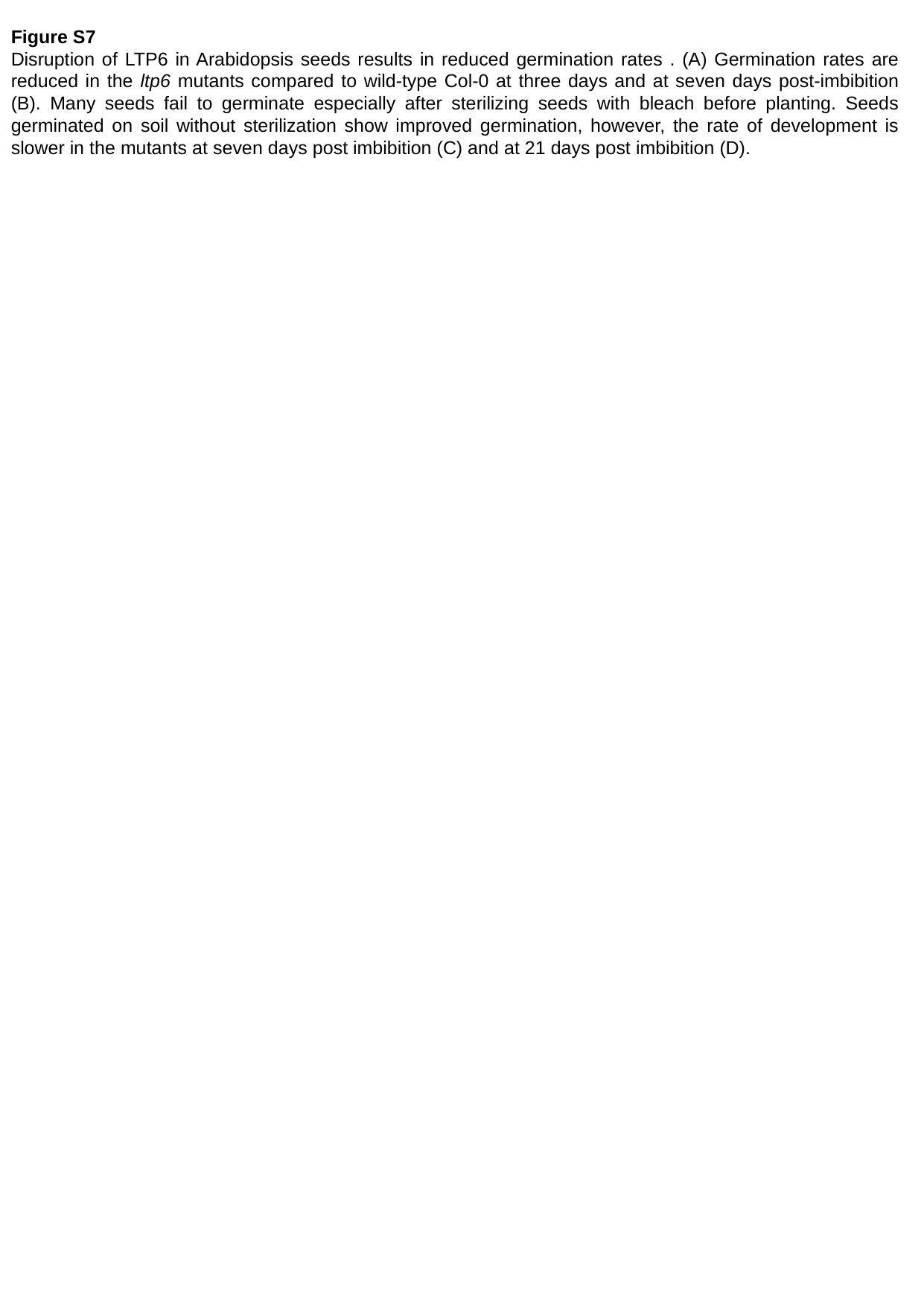

Figure S7
Disruption of LTP6 in Arabidopsis seeds results in reduced germination rates . (A) Germination rates are reduced in the ltp6 mutants compared to wild-type Col-0 at three days and at seven days post-imbibition (B). Many seeds fail to germinate especially after sterilizing seeds with bleach before planting. Seeds germinated on soil without sterilization show improved germination, however, the rate of development is slower in the mutants at seven days post imbibition (C) and at 21 days post imbibition (D).

## Slide 10
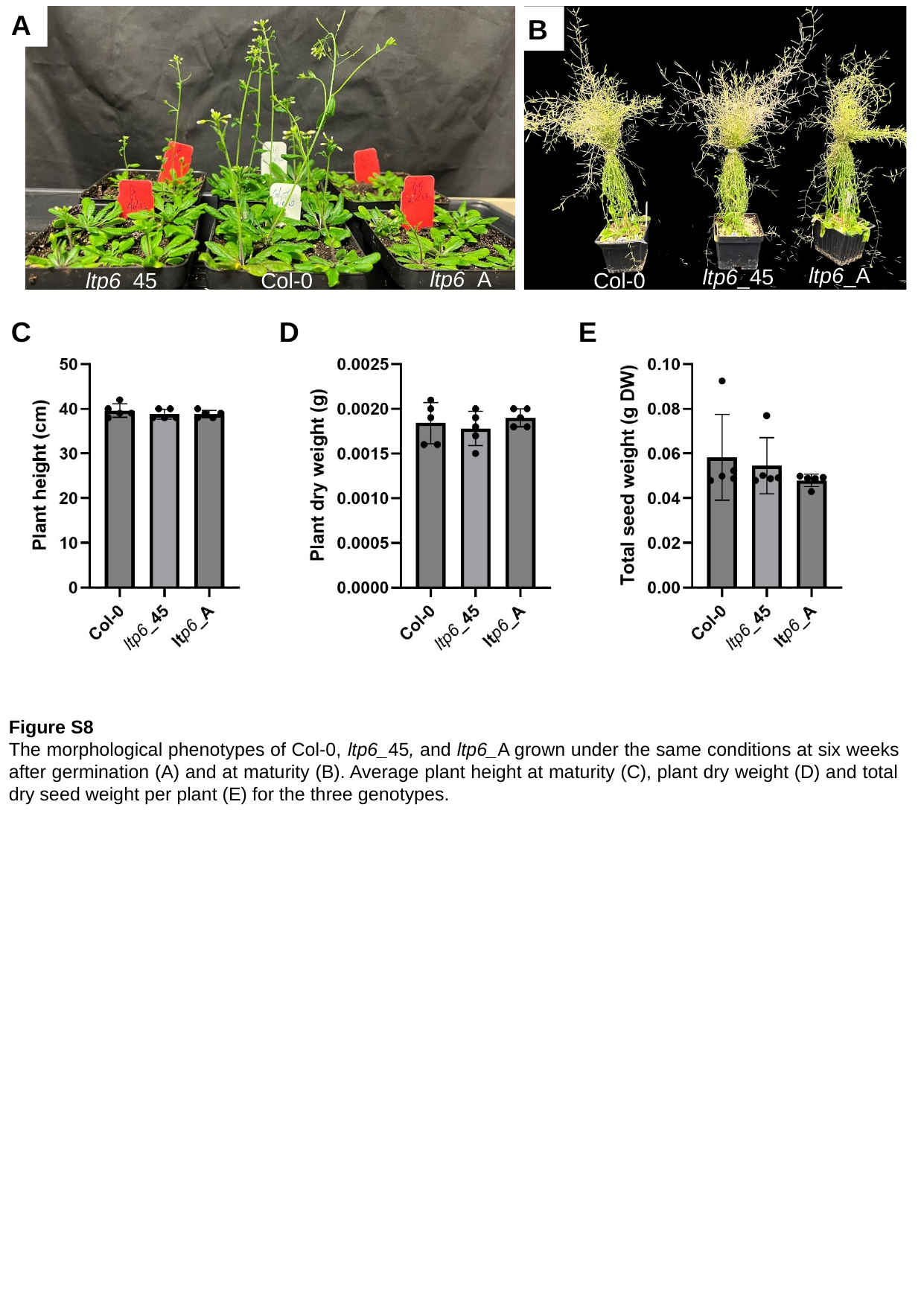

A
B
ltp6_A
ltp6_45
ltp6_A
ltp6_45
Col-0
Col-0
C
D
E
ltp6_A
ltp6_45
Col-0
Figure S8
The morphological phenotypes of Col-0, ltp6_45, and ltp6_A grown under the same conditions at six weeks after germination (A) and at maturity (B). Average plant height at maturity (C), plant dry weight (D) and total dry seed weight per plant (E) for the three genotypes.

## Slide 11
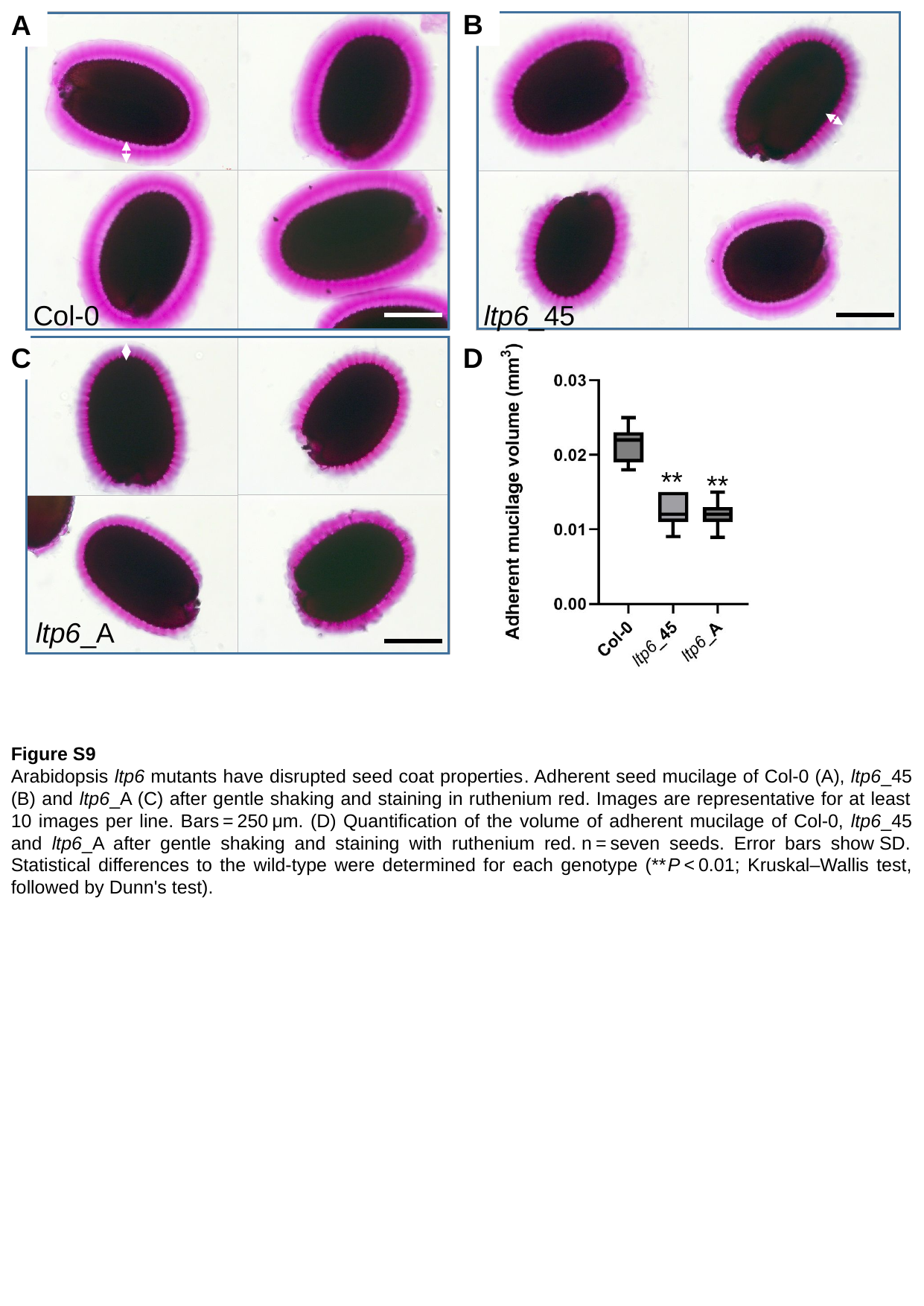

B
A
Col-0
ltp6_45
C
D
**
**
ltp6_A
Figure S9
Arabidopsis ltp6 mutants have disrupted seed coat properties. Adherent seed mucilage of Col-0 (A), ltp6_45 (B) and ltp6_A (C) after gentle shaking and staining in ruthenium red. Images are representative for at least 10 images per line. Bars = 250 μm. (D) Quantification of the volume of adherent mucilage of Col-0, ltp6_45 and ltp6_A after gentle shaking and staining with ruthenium red. n = seven seeds. Error bars show SD. Statistical differences to the wild-type were determined for each genotype (**P < 0.01; Kruskal–Wallis test, followed by Dunn's test).

## Slide 12
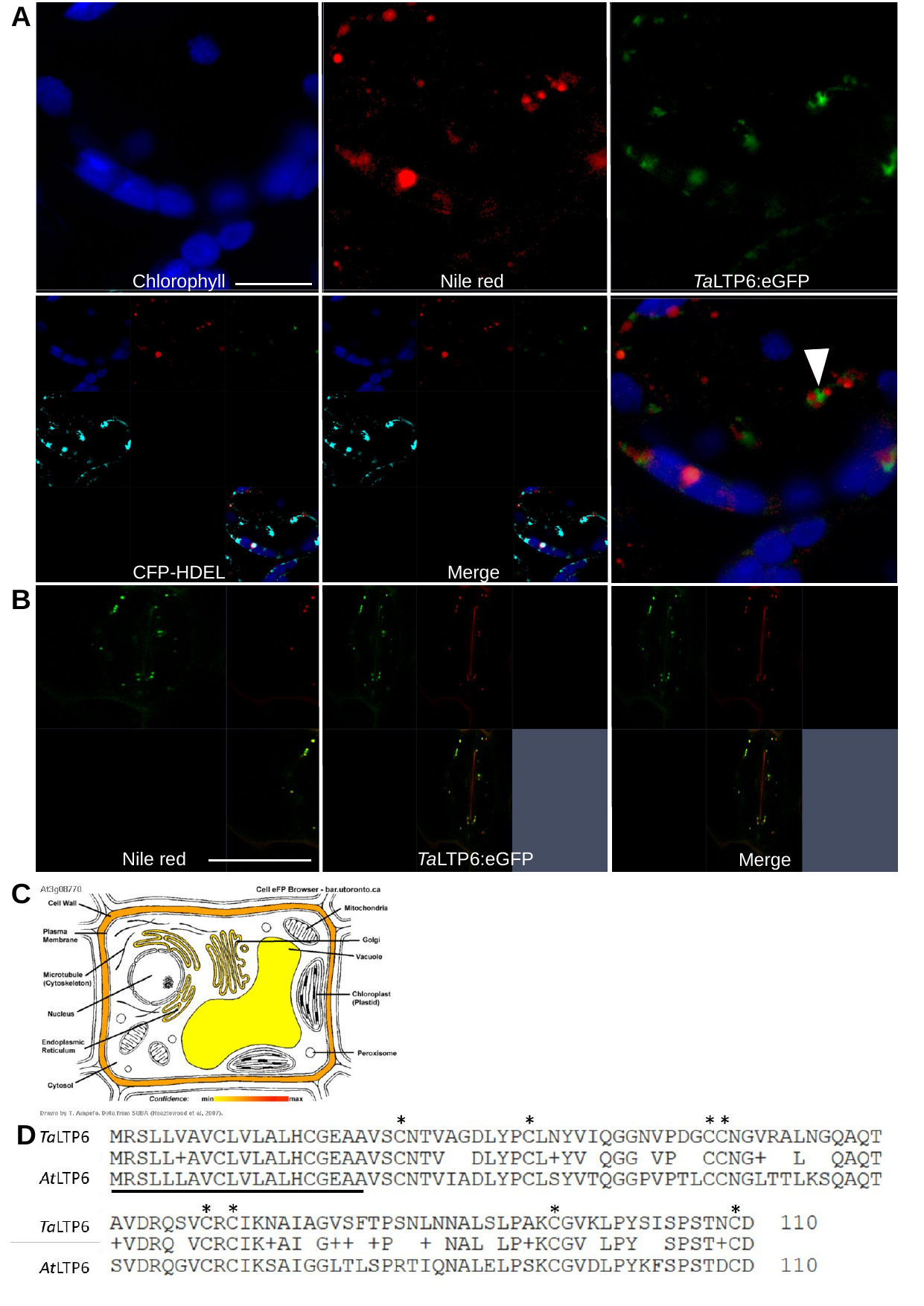

A
Chlorophyll
Nile red
TaLTP6:eGFP
CFP-HDEL
Merge
B
Nile red
TaLTP6:eGFP
Merge
C
D

## Slide 13
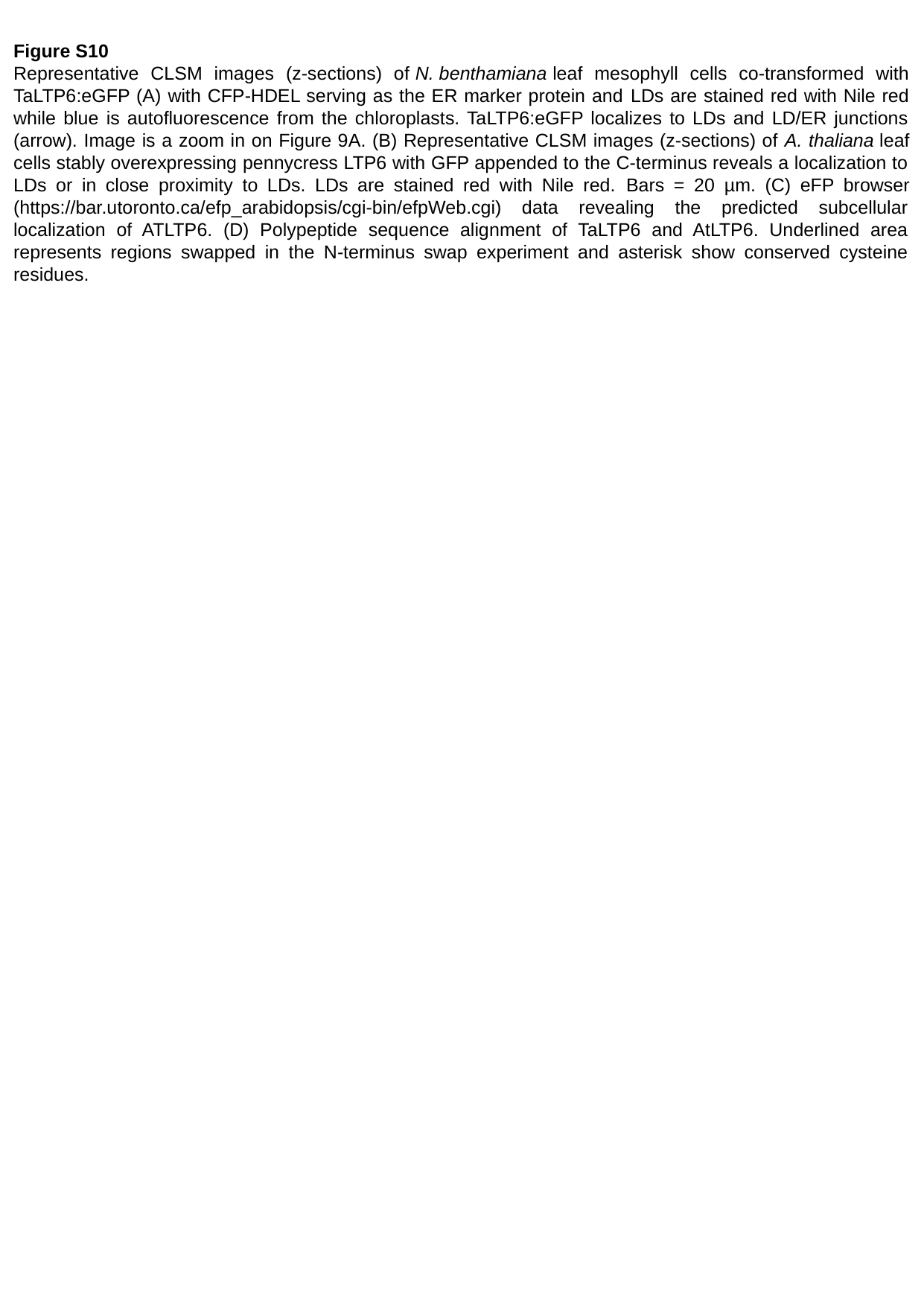

Figure S10
Representative CLSM images (z-sections) of N. benthamiana leaf mesophyll cells co-transformed with TaLTP6:eGFP (A) with CFP-HDEL serving as the ER marker protein and LDs are stained red with Nile red while blue is autofluorescence from the chloroplasts. TaLTP6:eGFP localizes to LDs and LD/ER junctions (arrow). Image is a zoom in on Figure 9A. (B) Representative CLSM images (z-sections) of A. thaliana leaf cells stably overexpressing pennycress LTP6 with GFP appended to the C-terminus reveals a localization to LDs or in close proximity to LDs. LDs are stained red with Nile red. Bars = 20 µm. (C) eFP browser (https://bar.utoronto.ca/efp_arabidopsis/cgi-bin/efpWeb.cgi) data revealing the predicted subcellular localization of ATLTP6. (D) Polypeptide sequence alignment of TaLTP6 and AtLTP6. Underlined area represents regions swapped in the N-terminus swap experiment and asterisk show conserved cysteine residues.

## Slide 14
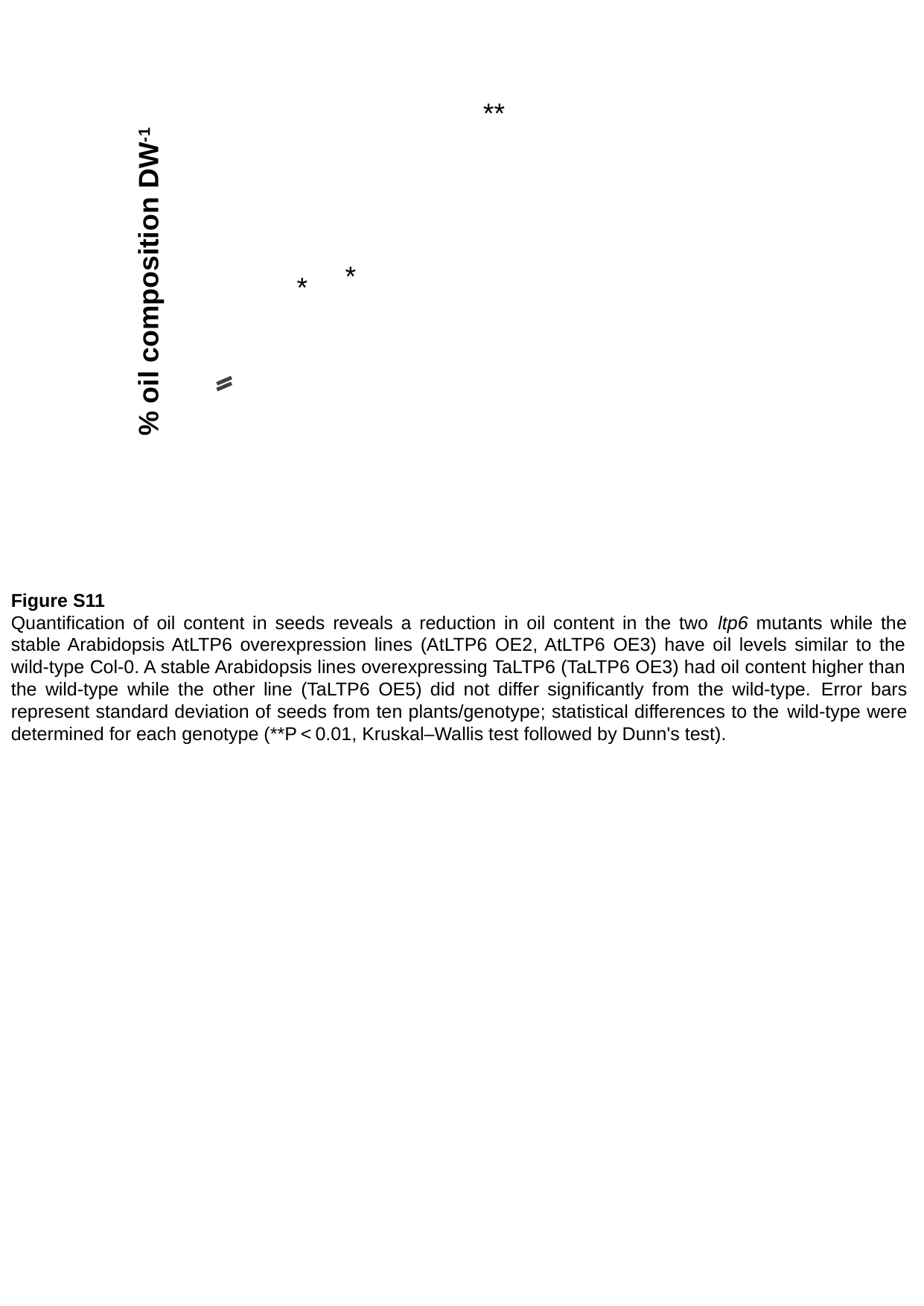

**
% oil composition DW-1
*
*
Figure S11
Quantification of oil content in seeds reveals a reduction in oil content in the two ltp6 mutants while the stable Arabidopsis AtLTP6 overexpression lines (AtLTP6 OE2, AtLTP6 OE3) have oil levels similar to the wild-type Col-0. A stable Arabidopsis lines overexpressing TaLTP6 (TaLTP6 OE3) had oil content higher than the wild-type while the other line (TaLTP6 OE5) did not differ significantly from the wild-type. Error bars represent standard deviation of seeds from ten plants/genotype; statistical differences to the wild-type were determined for each genotype (**P < 0.01, Kruskal–Wallis test followed by Dunn's test).

## Slide 15
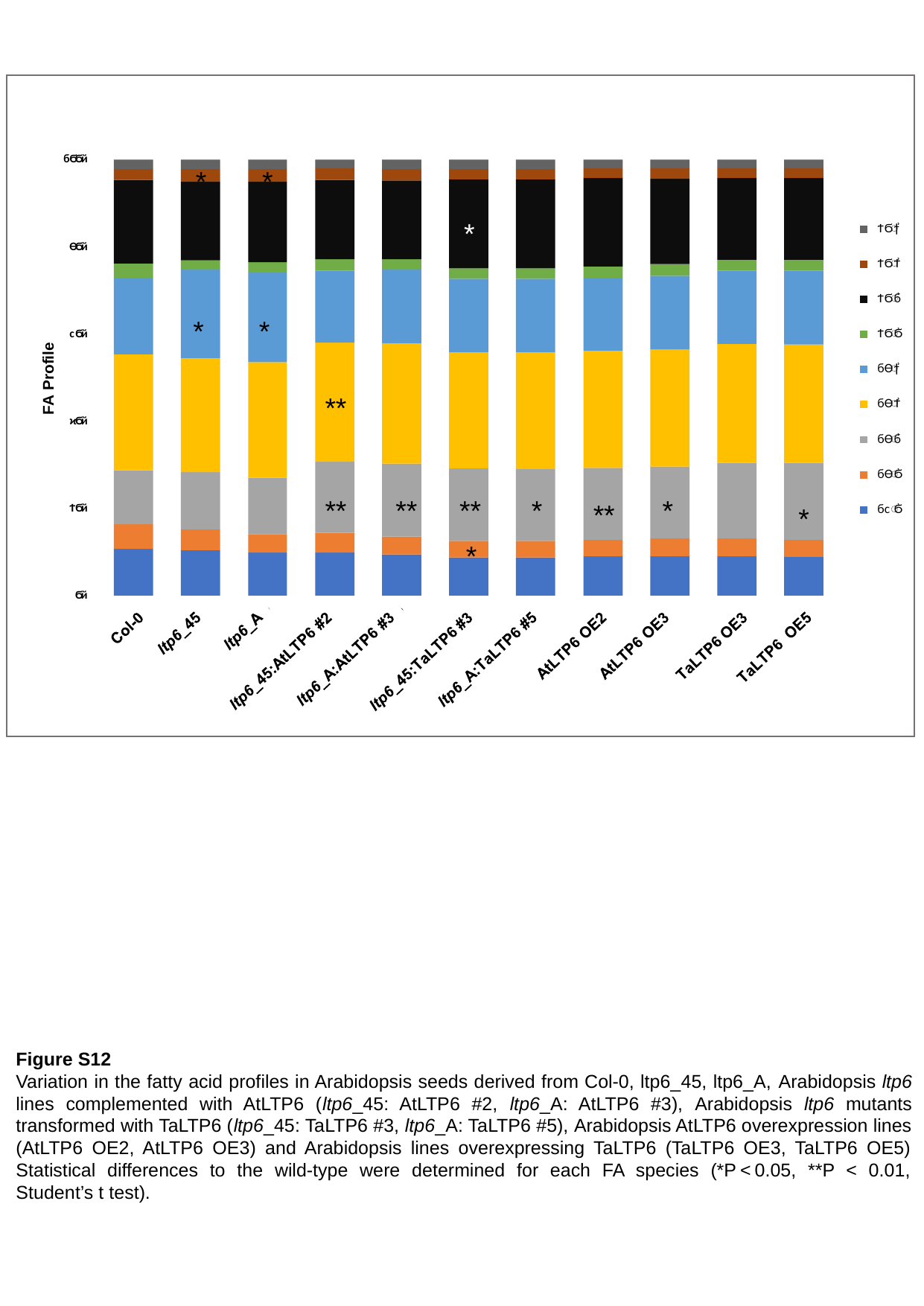

Figure S12
Variation in the fatty acid profiles in Arabidopsis seeds derived from Col-0, ltp6_45, ltp6_A, Arabidopsis ltp6 lines complemented with AtLTP6 (ltp6_45: AtLTP6 #2, ltp6_A: AtLTP6 #3), Arabidopsis ltp6 mutants transformed with TaLTP6 (ltp6_45: TaLTP6 #3, ltp6_A: TaLTP6 #5), Arabidopsis AtLTP6 overexpression lines (AtLTP6 OE2, AtLTP6 OE3) and Arabidopsis lines overexpressing TaLTP6 (TaLTP6 OE3, TaLTP6 OE5) Statistical differences to the wild-type were determined for each FA species (*P < 0.05, **P < 0.01, Student’s t test).

## Slide 16
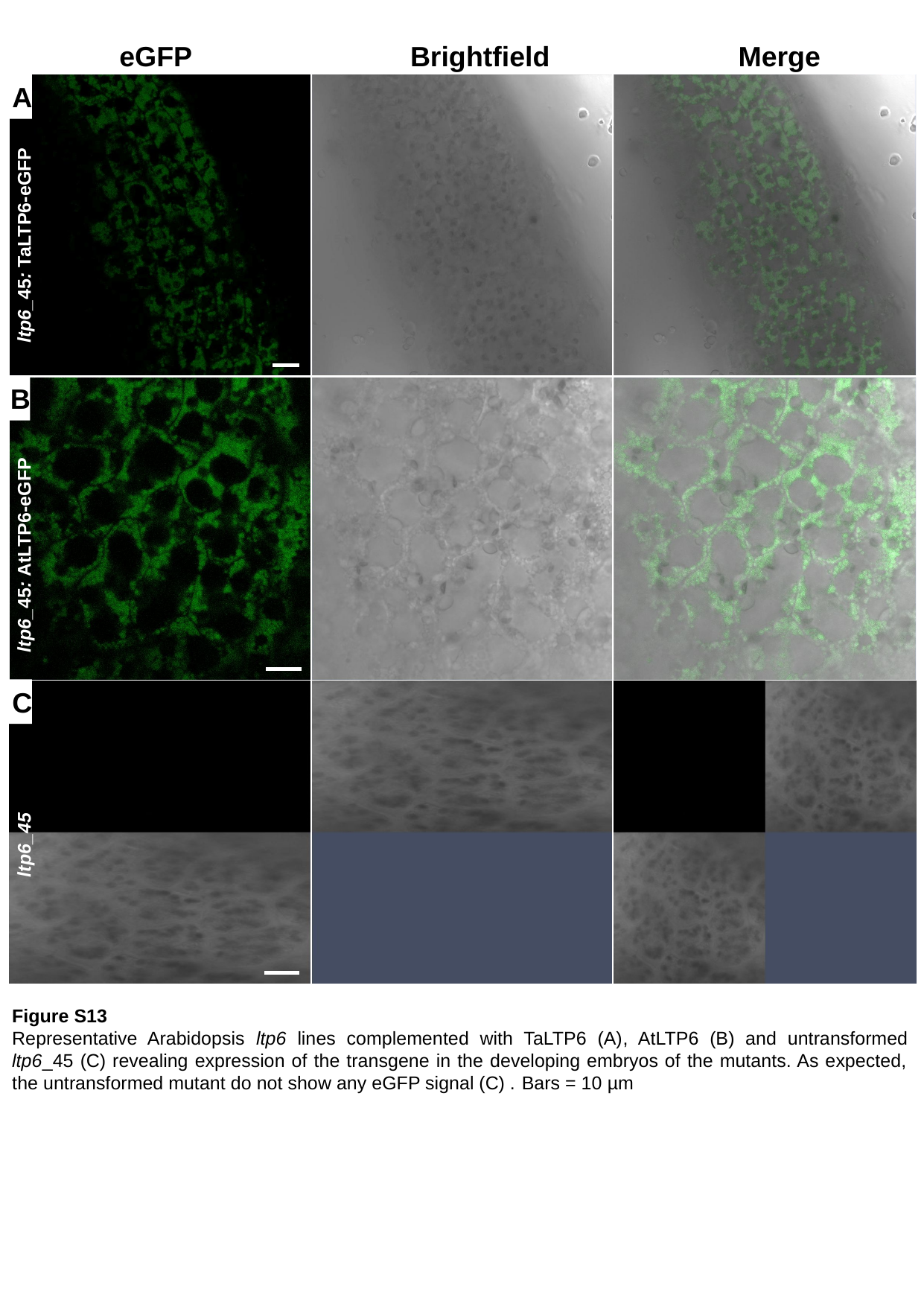

eGFP
Brightfield
Merge
A
ltp6_45: TaLTP6-eGFP
B
ltp6_45: AtLTP6-eGFP
C
ltp6_45
Merge
Figure S13
Representative Arabidopsis ltp6 lines complemented with TaLTP6 (A), AtLTP6 (B) and untransformed ltp6_45 (C) revealing expression of the transgene in the developing embryos of the mutants. As expected, the untransformed mutant do not show any eGFP signal (C) . Bars = 10 µm
Brightfield
