# Supplementary material for: Functional analysis of LIPID TRANSFER PROTEIN 6 (LTP6) in pennycress and Arabidopsis reveals divergent roles in oil storage and seed coat development
Source: Plant J. 2026 Jul 13;127(1):e71038. doi: 10.1111/tpj.71038 (PMC13363014; doi:10.1111/tpj.71038)
Supplement: Supplementary file 2 — Table S1. List of primers. [file TPJ-127-0-s001.docx]

Table S1: List of primers

| **Cloning primers** | **Forward 5’-3’** | **Reverse 5’-3’** |
| --- | --- | --- |
| TaLTP6 pMDC32/pDMC43 | GGGCCCGGCGCGCCAATGAGATCTCTCTTAGTGGCC | GGGCCCTTAATTAATCATTACCTGTCGCAGTTGG |
| TaLTP6 pMDC84 | GGGCCCTTAATTAAATGAGATCTCTCTTAGTGGCC | GGGCCCGGCGCGCCATTACCTGTCGCAGTTGGTG |
| TruncTaLTP6pMDC32/43 | GGGCCCGGCGCGCCAATGGCCGTGTCTTGCAACA | GGGCCCTTAATTAATCATTACCTGTCGCAGTTGG |
| TruncTaLTP6 pMDC84 | GGGCCCTTAATTAAATGGCCGTGTCTTGCAACA | GGGCCCGGCGCGCCATTACCTGTCGCAGTTGGTG |
| AtLTP6 pMDC32/pDMC43 | GGGCCCGGCGCGCCAATGAGATCTCTCTTATTAGCCGTGT | GGGCCCTTAATTAATCACTGGATACTGTCGCAGTC |
| AtLTP6 pMDC84 | GGGCCCTTAATTAAATGAGATCTCTCTTATTAGCCGTGT | GGGCCCGGCGCGCCACTGGATACTGTCGCAGTCAG |
| TruncAtLTP6pMDC32/43 | GGGCCCGGCGCGCCAATGGCAGCCGTGTCTTGCAA | GGGCCCTTAATTAATCACTGGATACTGTCGCAGTC |
| TruncatLTP6 pMDC84 | GGGCCCTTAATTAAATGGCAGCCGTGTCTTGCAA | GGGCCCGGCGCGCCACTGGATACTGTCGCAGTCAG |
| **Genotyping primers** | **Forward 5’-3’** | **Reverse 5’-3’** |
| \| SALK_043234LP \| \| --- \| \|  \| | GCCAGAATCGGGAGATGAGGCTTC | CACTGCGGTGAAGCAGCCG |
| SALK_120555 | AACTAGCAAACCAATGCCCTC | TTTTTCCTTTTGTCGACGTTG |
| **RT-PCR primers** | **Forward 5’-3’** | **Reverse 5’-3’** |
| AtLTP6 | TGCAACGGTCTCACAACACT | TCTGCTTGTCTCACTGTCGC |
| AtActin8 | GGTTTTCCCCAGTGTTGTTG | CTCCATGTCATCCCAGTTGC |
| TaLTP6 | GTGGCTGGAGACCTTTACCC | AGCAGGCAAAGACAGAGCAT |
| TaGAPDH | ACCGGTCTACAAACTCCCGT | TGGAAAGGTGCTTCCACAGC |
