# Supplementary material for: Functional analysis of LIPID TRANSFER PROTEIN 6 (LTP6) in pennycress and Arabidopsis reveals divergent roles in oil storage and seed coat development
Source: Plant J. 2026 Jul 13;127(1):e71038. doi: 10.1111/tpj.71038 (PMC13363014; doi:10.1111/tpj.71038)
Supplement: Supplementary file 3 — Table S2. Proteins identified in wild‐type Col‐0 microsome/membrane fraction. [file TPJ-127-0-s002.docx]

Table S2. Proteins identified in wild-type Col-0 microsome/membrane fraction.

| Gene | Description | Localization |
| --- | --- | --- |
| AT3G02090 | Mitochondrial-processing peptidase subunit beta (MPPBETA) | Mitochondrion, nucleolus, mitochondrial respiratory chain complex III, chloroplast, membrane, vacuole. |
| AT5G15090 | Arabidopsis thaliana voltage dependent anion channel 3 (VDAC3) | Mitochondrion, nucleolus, cell wall, chloroplast, membrane, vacuole. |
| AT2G07698 | ATP synthase | Mitochondrion. |
| AT1G71695 | Peroxidase superfamily protein | Cell wall, vacuole, membrane, plant-type cell wall. |
| AT5G08690 | ATP synthase alpha/beta family protein | Mitochondrion, nucleolus, mitochondrial respiratory chain complex I, chloroplast envelope, mitochondrial proton-transporting ATP synthase complex, catalytic core F. |
| AT5G44120 | RmlC-like cupins superfamily protein (CRA1) | Endomembrane system. |
| AT1G68560 | Alpha-xylosidase 1 (XYL1) | Apoplast, cell wall, chloroplast, plant-type cell wall. |
| AT3G01570 | Oleosin family protein | Monolayer-surrounded lipid storage body, integral to membrane, membrane. |
| AT1G44575 | Chlorophyll A-B binding family protein  (Chlorophyll A-B BP) | Thylakoid, chloroplast thylakoid membrane, chloroplast, PSII associated light-harvesting complex II, membrane. |
| AT5G40770 | Prohibitin 3 (PHB3) | Mitochondrion, nucleolus, mitochondrial respiratory chain complex I, chloroplast, membrane, vacuole. |
| AT4G16160 | Mitochondrial import inner membrane translocase subunit Tim17/Tim22/Tim23 family protein  (ATOEP16-2) | Plastid outer membrane, mitochondrial inner membrane presequence translocase complex. |
| AT3G08580 | ADP/ATP carrier 1 (AAC1) | Mitochondrion, nucleolus, chloroplast, membrane, vacuole, cell wall. |
| AT1G54860 | Glycoprotein membrane precursor GPI-anchored (GPI-anchored) | Extra-cellular space, membrane. |
| AT3G11050 | Ferritin 2 (ATFER2) | Chloroplasts |
| AT1G51980 | Insulinase (Peptidase family M16) protein | Mitochondrion, plasma membrane, plastid, mitochondrial respiratory chain complex III, membrane |
